# Supplementary material for: Large thermoelectric power factor from crystal symmetry-protected non-bonding orbital in half-Heuslers
Source: Nat Commun. 2018 Apr 30;9:1721. doi: 10.1038/s41467-018-03866-w (PMC5928102; doi:10.1038/s41467-018-03866-w)
Supplement: Supplementary file 1 — Supplementary Information [file 41467_2018_3866_MOESM1_ESM.pdf]

Supplementary Information

Zhou et al.

Supplementary Figure Legends

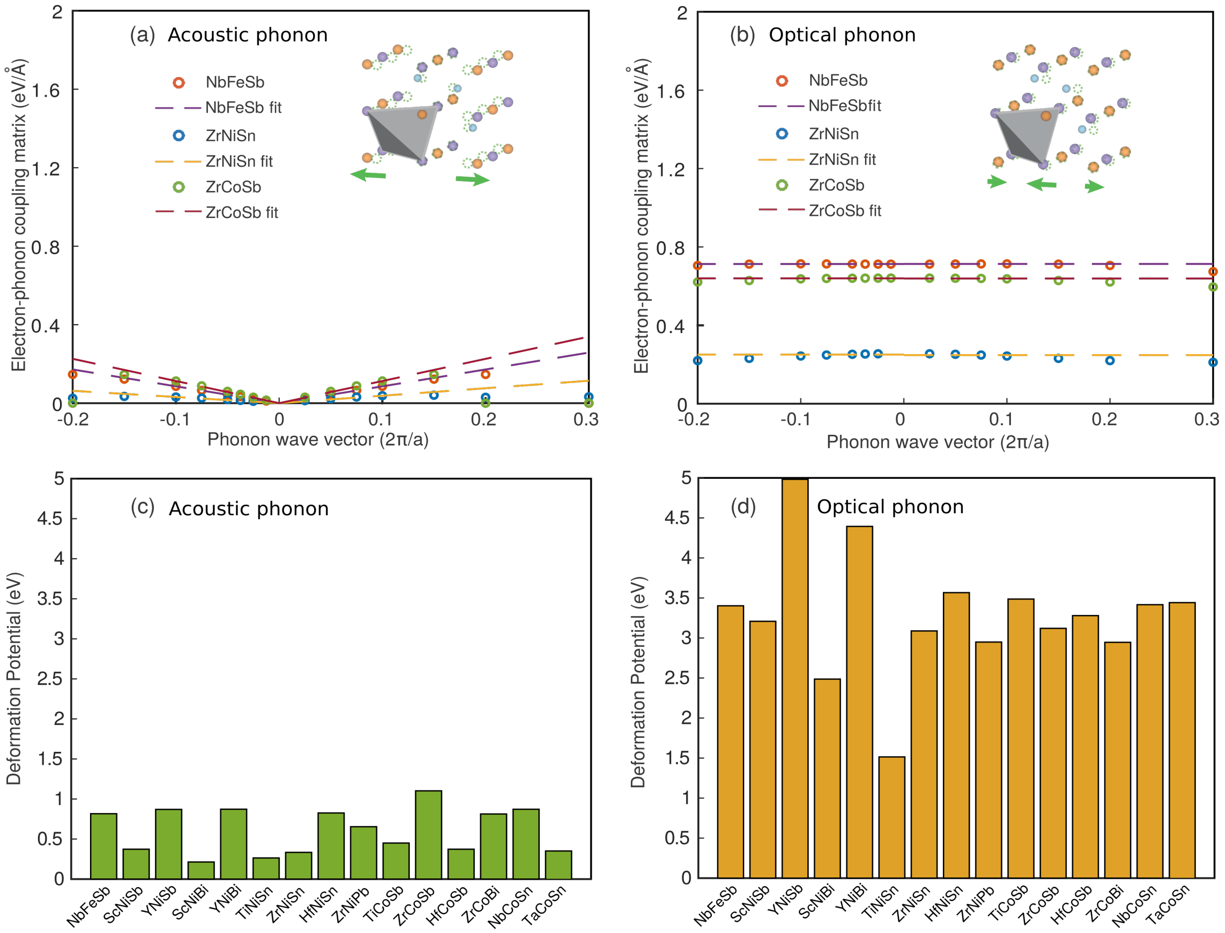

Supplementary Figure 1. Electron-phonon interaction and deformation potential for valence band. (a)-(b) Electron-phonon coupling (EPC) matrix along high symmetry lines for valence band edge states. For NbFeSb and ZrCoSb the initial electron state is located at L point while for ZrNiSn it is at Γ point. (c)-(d) Averaged deformation potential for acoustic and optical phonons. Due to the degenerate feature of valence band edge states, the EPC's in (a)-(b) is defined to an averaged value according to $\overline{D}(\mathbf{k},\mathbf{k}+\mathbf{q}) = \sqrt{\sum_{\mathrm{n,m}=1}^{N_\mathrm{d}}|D_{\mathrm{nm}}(\mathbf{k},\mathbf{k}+\mathbf{q})|^2/N_\mathrm{d}^2}$[10], where N_d is the degeneracy number, and n (m) denotes the band index that sums over all the degenerate states at point **k**. One difference in optical deformation potential between conduction bands and valence bands is that, in conduction band, EPC matrix goes to zero as phonon wave vector approaches zero which is dictated by symmetry, while in valence band, there is no symmetry to constrain its value and thus it is nearly a constant as normally expected[3]. By dividing this number by one fifth of the Brillouin zone size, characteristic of the typical range of thermally excited electrons (~5 k_BT), we obtain optical deformation potentials also in unit of energy. In general, for the valence band, the acoustic phonon deformation potentials (0.5 – 1 eV) are much smaller than optical ones (2.5 – 4 eV), which is consistent with our results for conduction band as shown in Fig. 1c-d.

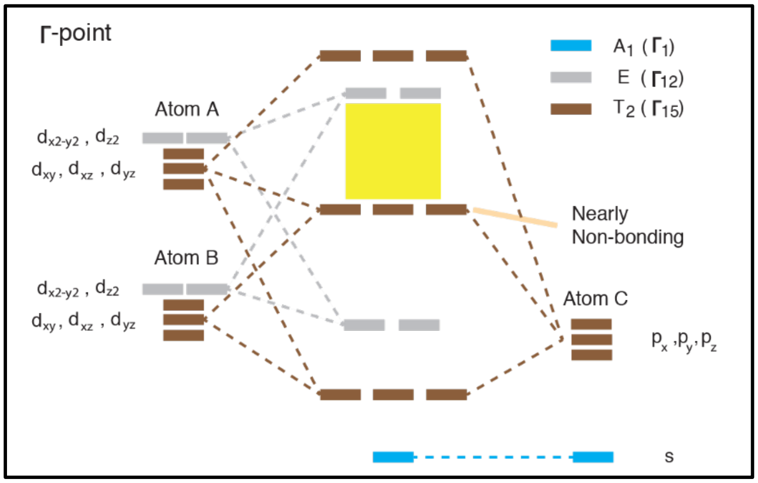

Supplementary Figure 2. Crystal orbital diagram for a prototypical half-Heusler semiconductor according to the point group symmetry T_d at Γ point. The region highlighted in yellow denotes the band gap. Note that the band edge state (triply degenerate) as characterized by representation T_2 has energy close to the atomic orbitals from atom A, and is nearly non-bonding. The predominant contribution from atom A can also be seen from the projected density of states plot in the example of ZrNiSn (Fig. 2d). The detailed derivation is given in Supplementary Note 4.

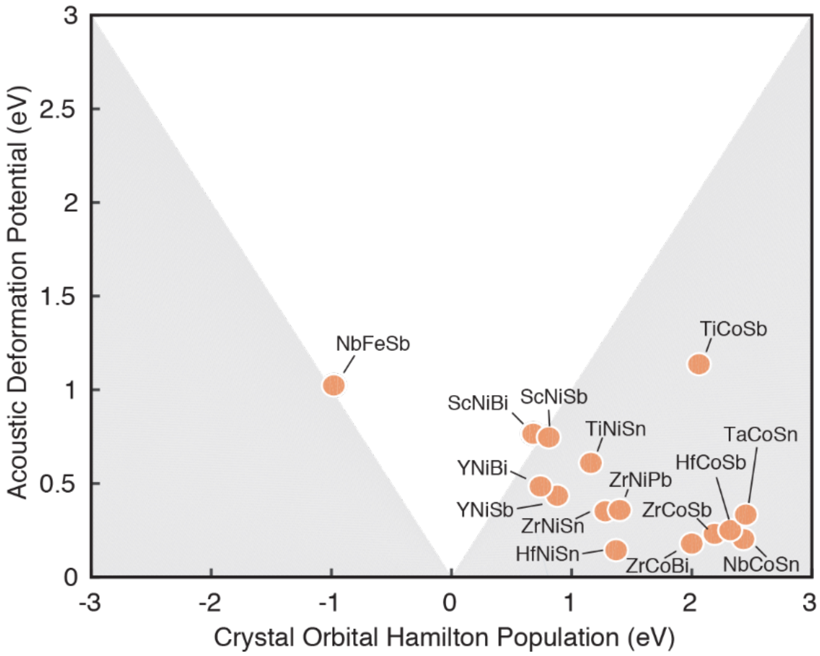

Supplementary Figure 3. Comparison between acoustic deformation potentials Ξ (for conduction band edge state) of half-Heusler materials and their calculated crystal orbital Hamilton population (COHP) values (at conduction band edge, see Supplementary Note 5). Positive COHP indicates anti-bonding-like interactions, while negative values suggest bonding character. The shaded area marks the region where $\Xi \leq \mathrm{COHP}$ is satisfied. The fact that all half-Heuslers fall within this region demonstrates that indeed the small acoustic deformation potential generally found in this material system can be quantified by their vanishing bonding or anti-bonding interactions, which also suggests the COHP as a useful quantity for estimating the strength of electron-acoustic phonon coupling.

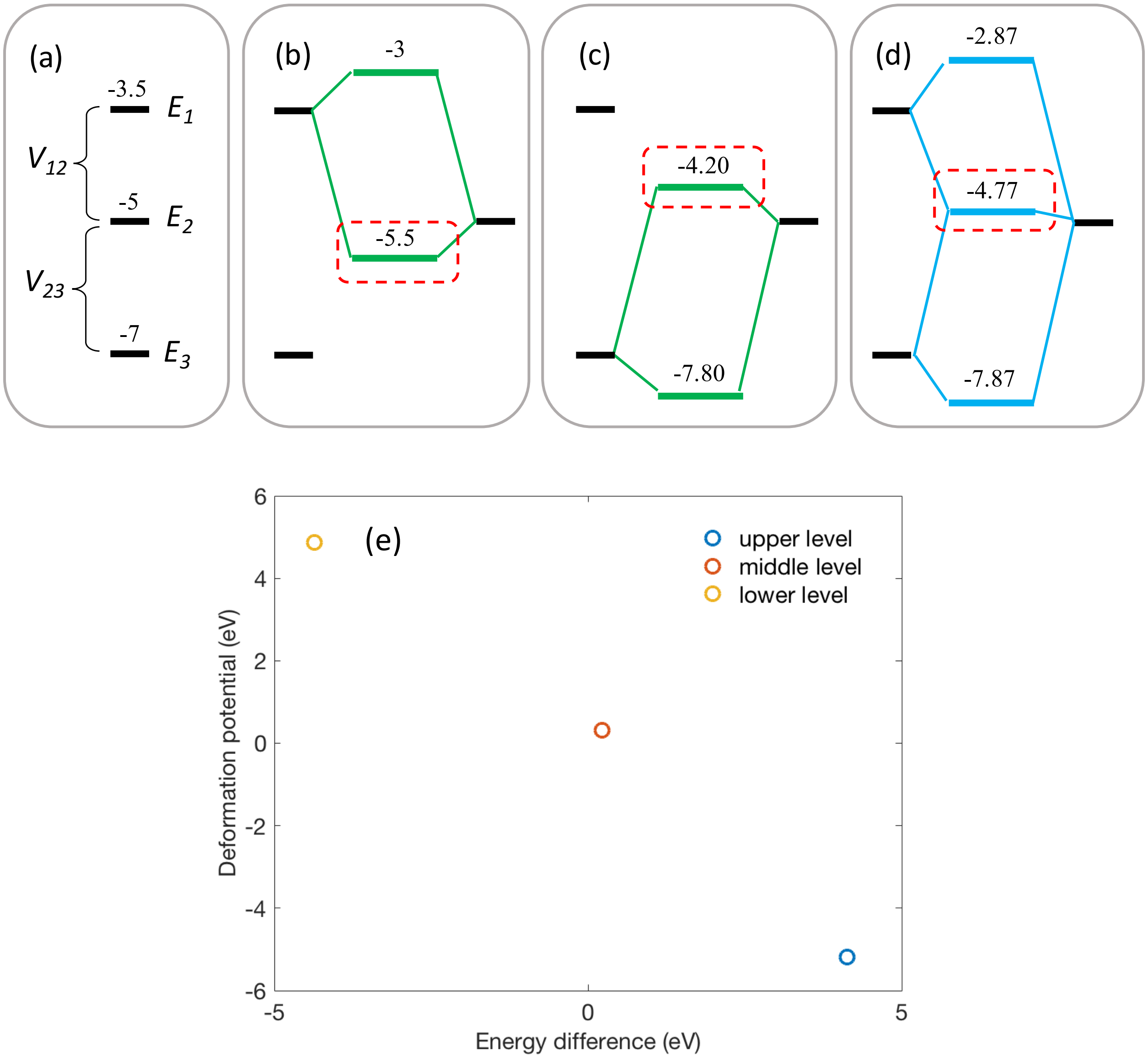

Supplementary Figure 4. (a) Schematic of the starting energy levels and their orbital interactions; (b) Orbital diagram if only interaction between state 1 and state 2 is turned on; (c) Orbital diagram if only interaction between state 2 and state 3 is turned on; (d) Orbital diagram if both interactions between 1 and 2, as well as 2 and 3, are turned on; (e) Deformation potentials compared with energy level shift in a three-level tight-binding model. In (a)-(d), the energy value of each state has been labelled correspondingly (before and after interactions).

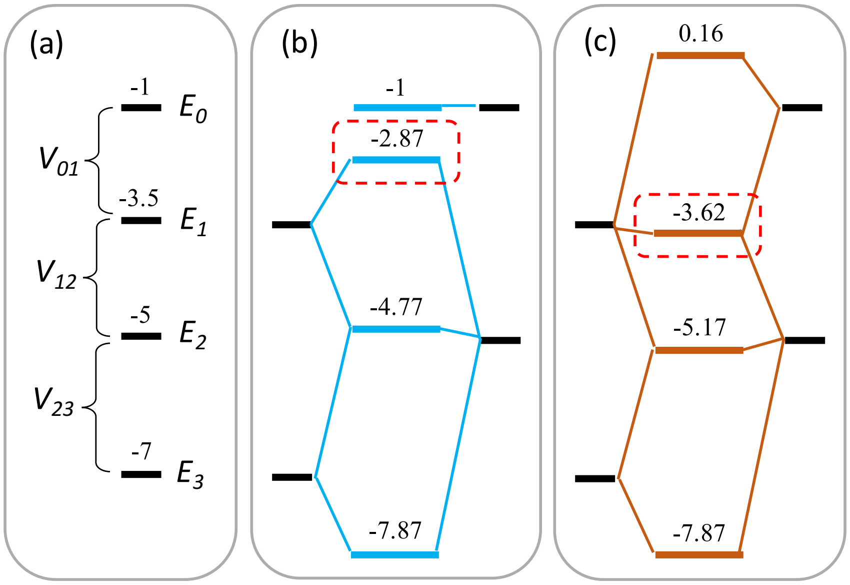

Supplementary Figure 5. (a) Schematic of the starting energy levels in a three-level system with an additional level; (b) Orbital diagram if interaction between state 0 and state 1 is turned off; (c) Orbital diagram if interaction between state 0 and state 1 is turned on.

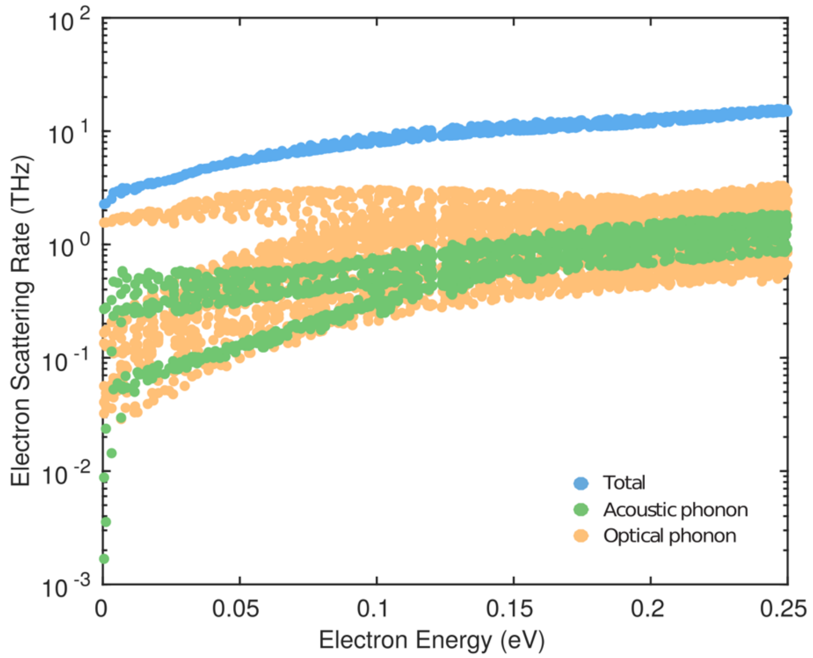

Supplementary Figure 6. Electron scattering rates decomposed into contributions from different branches for ZrNiSn at room temperature with a carrier concentration of 10^{21} cm^{-3}.

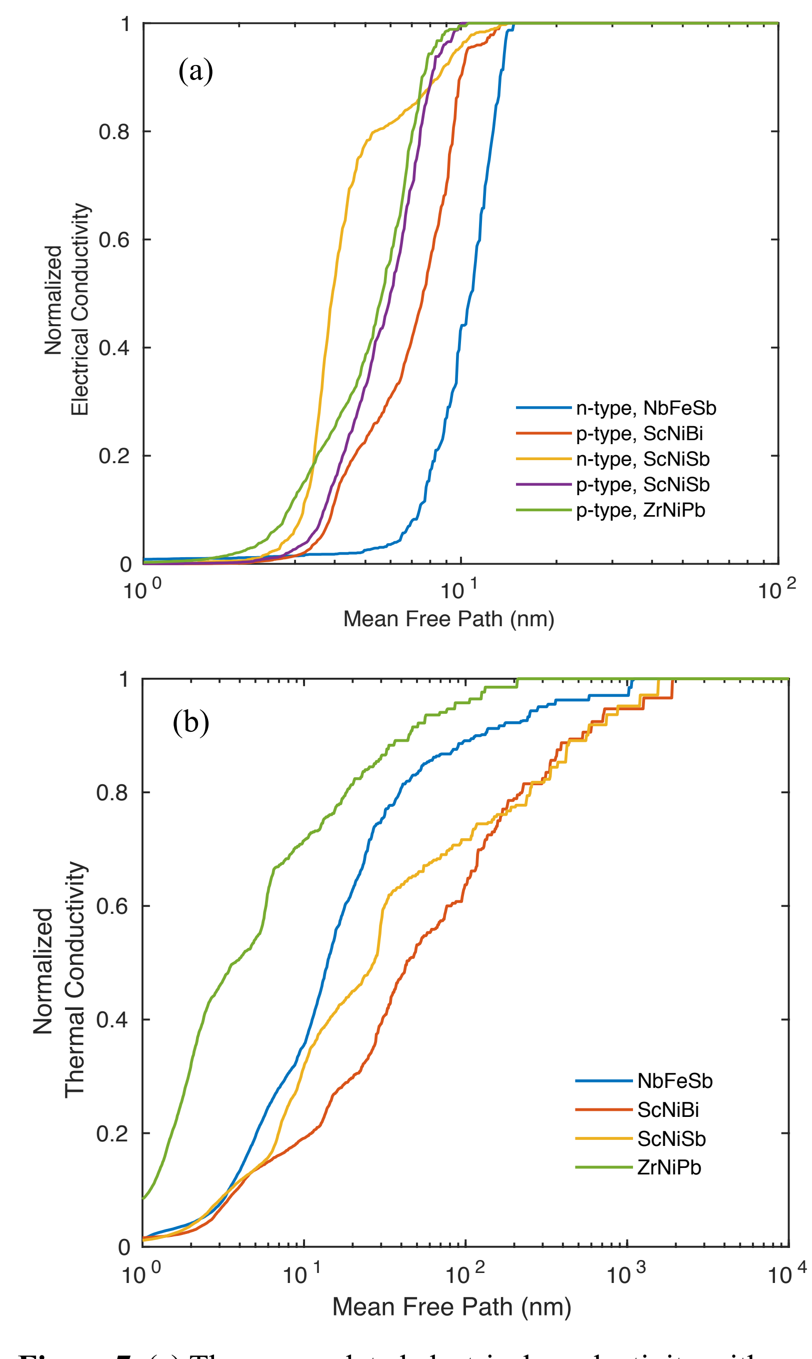

Supplementary Figure 7. (a) The accumulated electrical conductivity with respect to the electron mean free path, and (b) the accumulated thermal conductivity with respect to the phonon mean free path, for select half-Heusler compounds at 1000K.

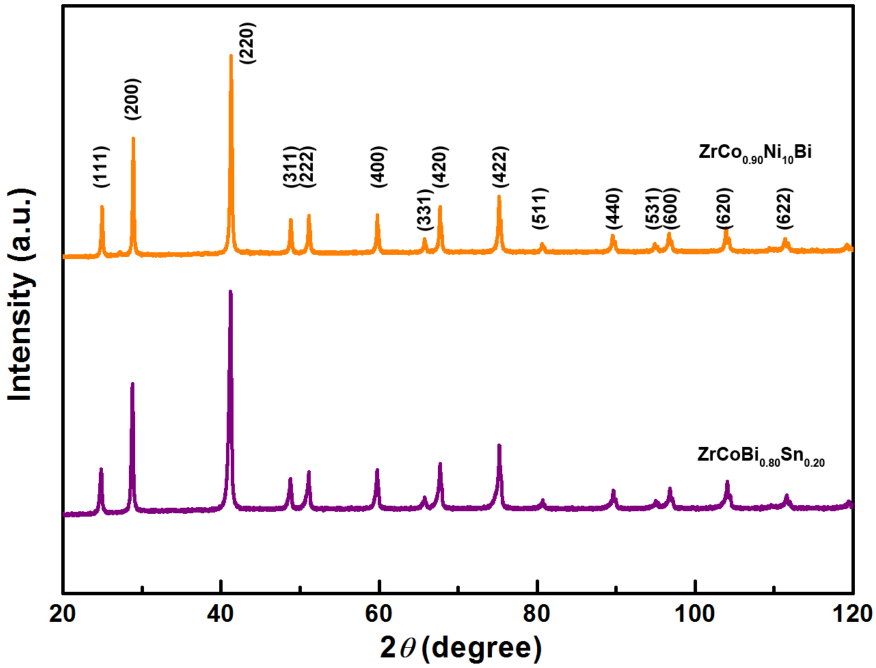

Supplementary Figure 8. X-ray diffraction (XRD) patterns of $ZrCo_{0.90}Ni_{0.10}Bi$ and $ZrCoBi_{0.80}Sn_{0.20}$ samples. Both of the compositions show half-Heusler phase.

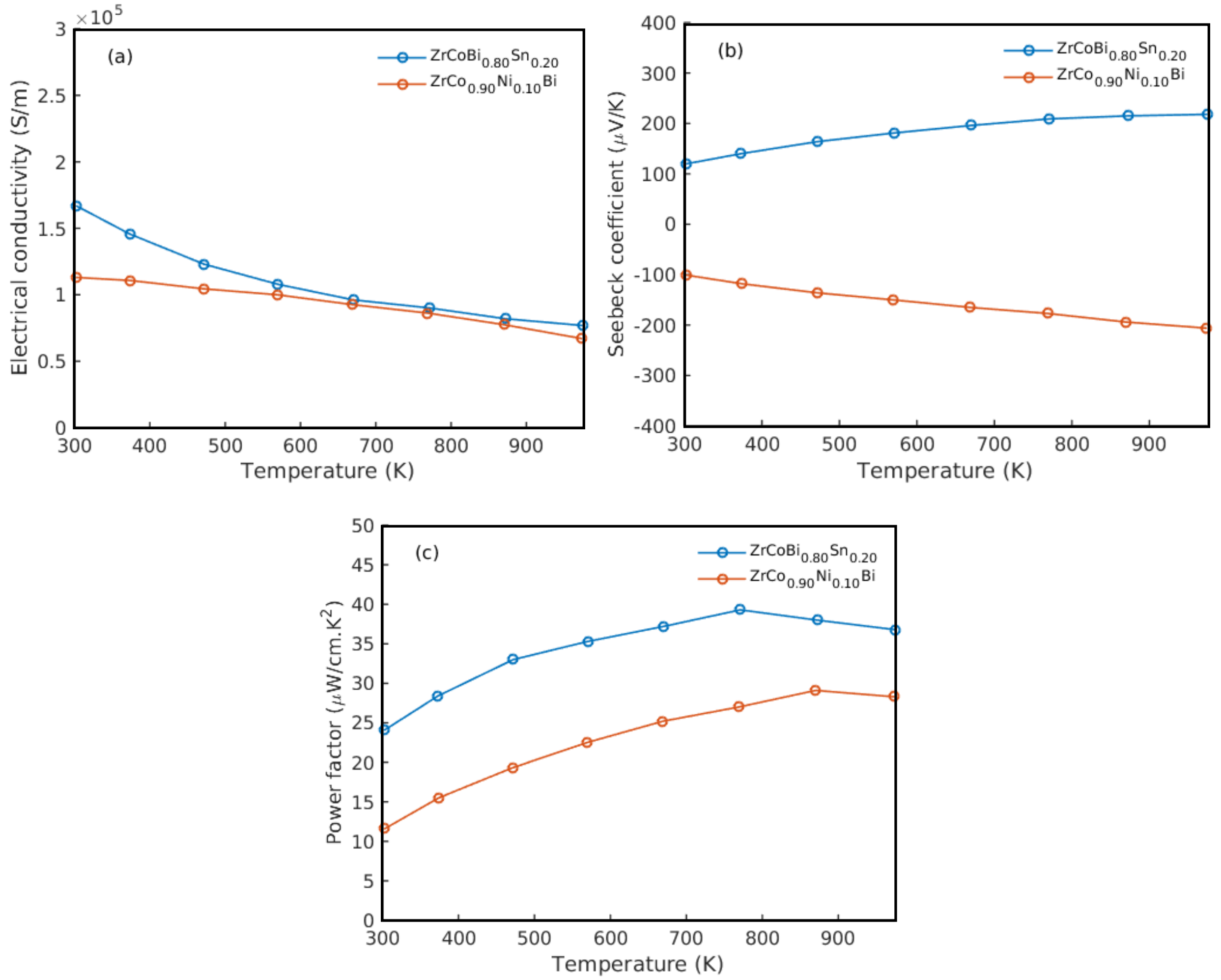

Supplementary Figure 9. Measured electrical transport property dependence on temperature for $ZrCo_{0.90}Ni_{0.10}Bi$ and $ZrCoBi_{0.80}Sn_{0.20}$ samples. (a) Electrical conductivity, (b) Seebeck coefficient, and (c) power factor are shown with temperature from 300K to 973K.

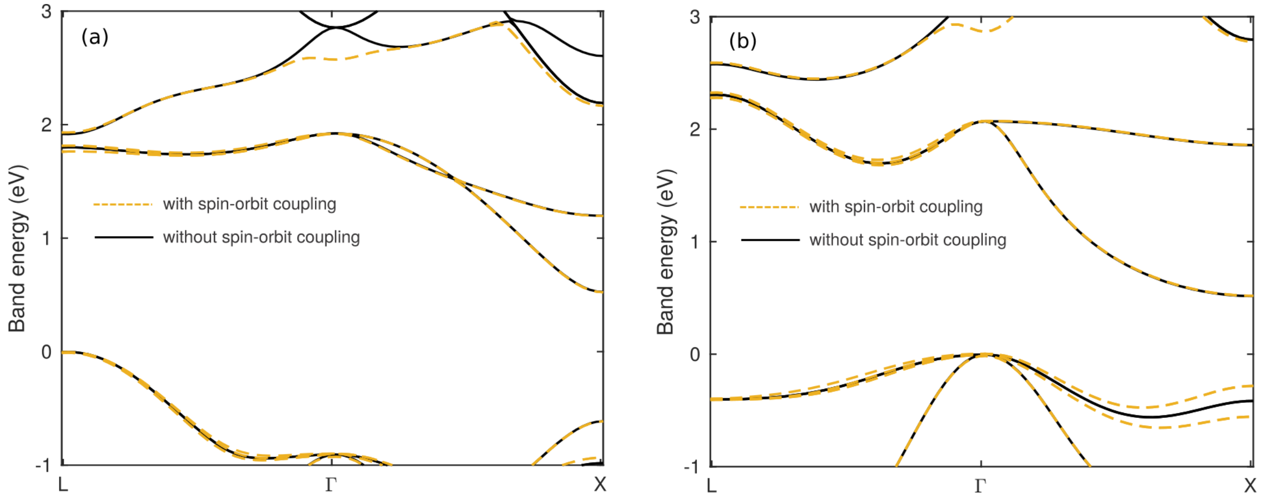

Supplementary Figure 10. Band structures near the band edge with and without the spin-orbit couplings (SOC). Results for (a) NbFeSb, and (b) ZrNiSn have been shown. The results without SOC are obtained using PAW pseudopotentials without relativistic corrections, while the results including SOC are obtained using fully-relativistic PAW pseudopotentials[66].

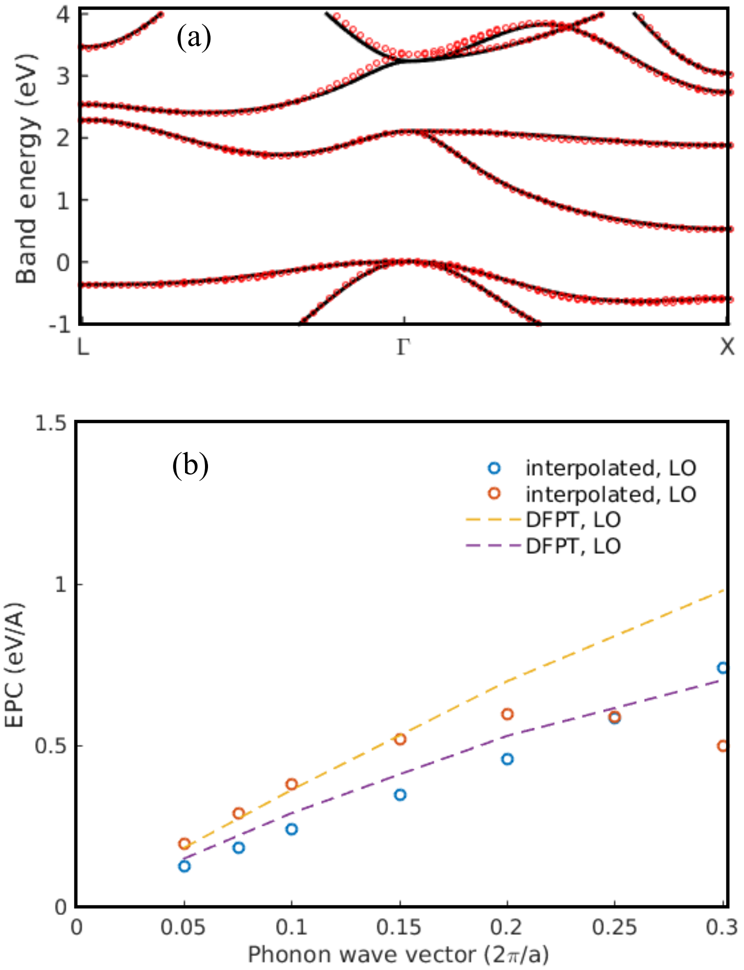

Supplementary Figure 11. Comparisons between direct first principles calculations and electron-phonon Wannier interpolations. (a) Band structure comparison between first principles calculation and interpolated result for ZrNiSn. The black lines are from first principles while the red dots are the interpolated results. (b) Electron-phonon coupling matrix comparison between first principles calculation and interpolated result for ZrNiSn. The dashed lines are from first principles while the dots are interpolated results. The initial electron is taken to be at the conduction band edge, while the phonon wave vector varies along the $\Gamma - X$ direction.

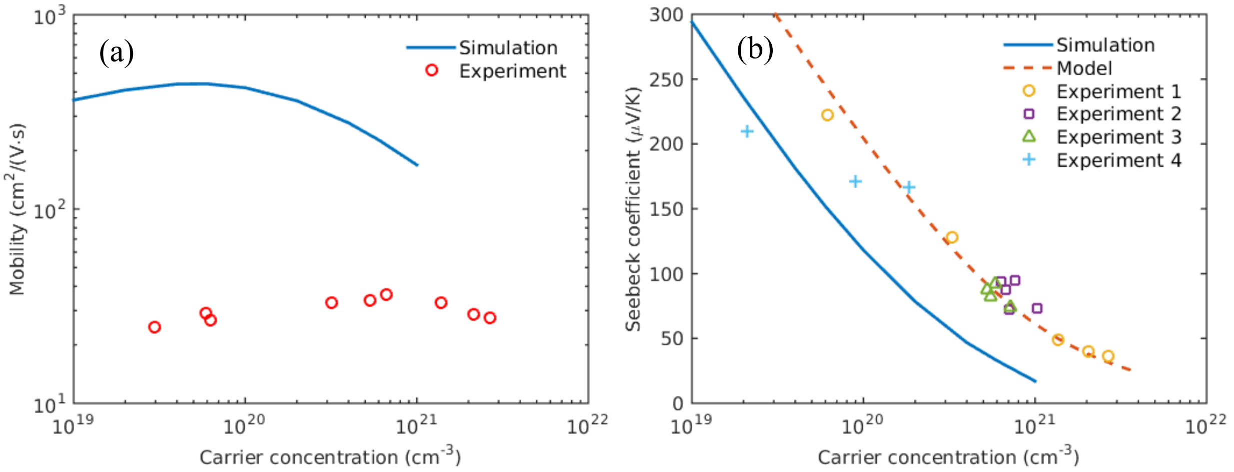

Supplementary Figure 12. Calculated (a) mobility and (b) Seebeck coefficient with different carrier concentrations compared to experiments at room temperature in ZrNiSn. The experimental data for mobility are taken from Ref.[21]. The experimental data sets 1-4 for Seebeck coefficient are taken from Ref.[21], Ref.[67], Ref.[23] and Ref.[24], respectively.

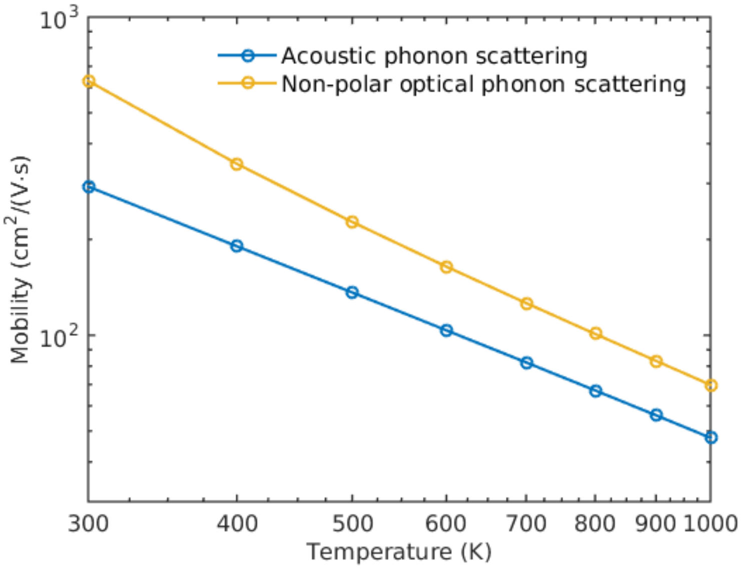

Supplementary Figure 13. Electron mobility as a function of temperature considering either only acoustic phonon scattering or non-polar optical phonon scattering, assuming single parabolic band. The effective mass is taken to be $1\mathrm{m}_e$ and the optical deformation potential is assumed to be constant ($5\mathrm{eV}/\mathrm{Å}$).

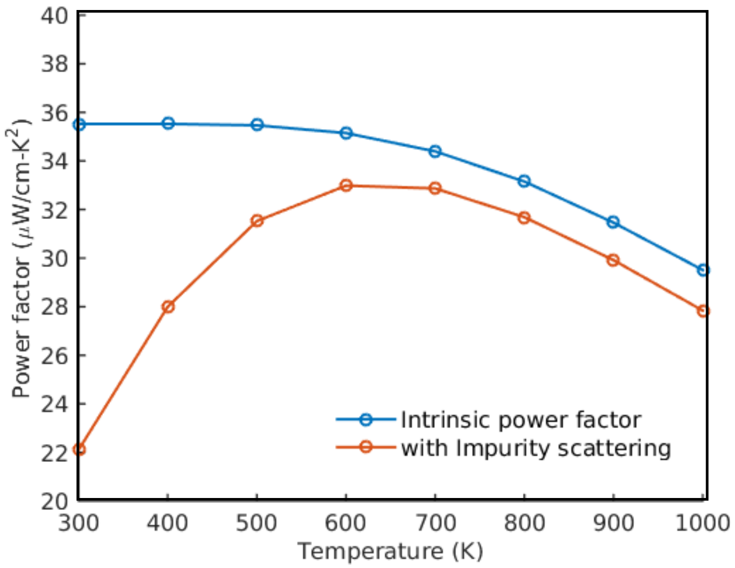

Supplementary Figure 14. Power factor with respect to temperature considering both acoustic phonon scattering and impurity scattering, assuming single parabolic band. The effective mass is taken to be $1m_e$ and the acoustic deformation potential is 5eV. Electron-impurity scattering is added according to the description in Supplementary Note 1 with an effective density of impurity just to illustrate its effect on the temperature dependence.

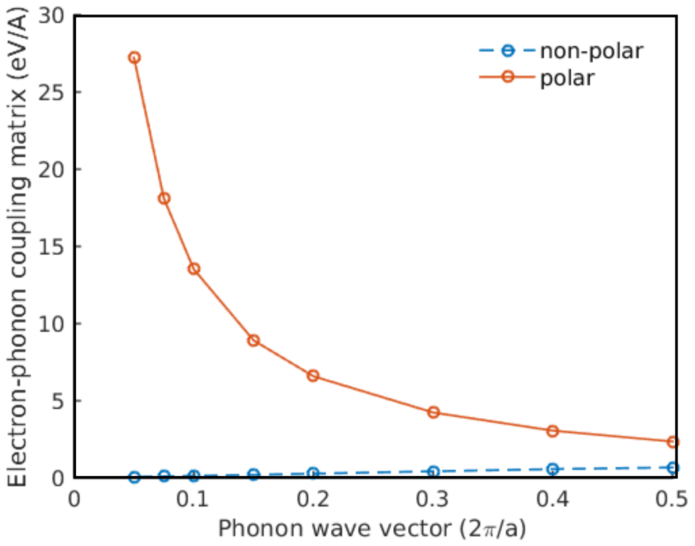

Supplementary Figure 15. Electron-phonon coupling matrix for the longitudinal optical phonon mode with and without the polar interactions. The initial electron is taken to be at the conduction band edge, and the phonon wave vector varies along the $\Gamma - X$ direction.

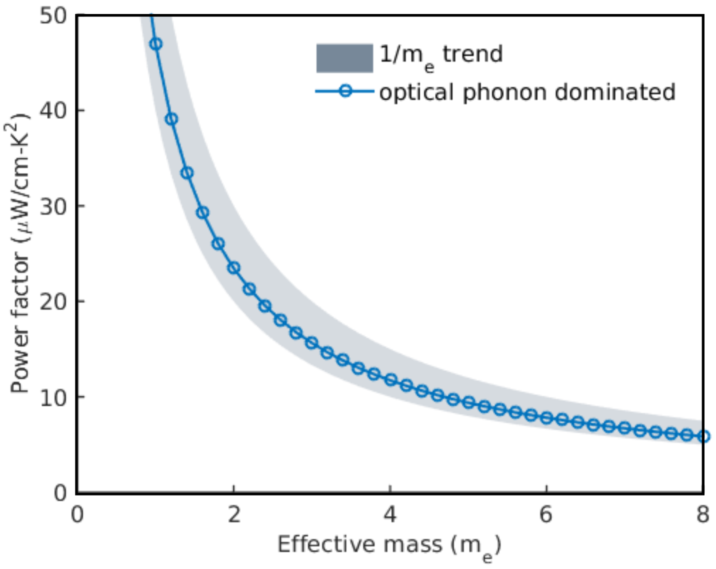

Supplementary Figure 16. Power factor with respect to effective mass considering only electron scatterings by non-polar optical phonons. The shaded region delineates the $1/m^*$ trend with upper and lower bound, showing that the optical phonon dominated transport with constant optical deformation potential can also be well characterized by this trend.

Supplementary Table 1. Calculated lattice constants for presented materials.

Material	Lattice constant (nm)
**NbFeSb**	0.600
**ScNiSb**	0.617
**YNiSb**	0.640
**ScNiBi**	0.629
**YNiBi**	0.650
**TiNiSn**	0.600
**ZrNiSn**	0.618
**HfNiSn**	0.615
**ZrNiPb**	0.629
**TiCoSb**	0.596
**ZrCoSb**	0.614
**HfCoSb**	0.611
**ZrCoBi**	0.624
**NbCoSn**	0.599
**TaCoSn**	0.600

Supplementary Table 2. Electron and phonon mesh densities (they are set to be the same) used in electron transport calculations to ensure convergence for the electron scattering rates.

Material	Electron (phonon) mesh<br>n-type / p-type
**NbFeSb**	80×80×80 / 60×60×60
**ScNiSb**	60×60×60 / 60×60×60
**YNiSb**	60×60×60 / 60×60×60
**ScNiBi**	60×60×60 / 60×60×60
**YNiBi**	60×60×60 / 60×60×60
**TiNiSn**	60×60×60 / 40×40×40
**ZrNiSn**	60×60×60 / 60×60×60
**HfNiSn**	60×60×60 / 60×60×60
**ZrNiPb**	60×60×60 / 60×60×60
**TiCoSb**	60×60×60 / 40×40×40
**ZrCoSb**	60×60×60 / 40×40×40
**HfCoSb**	60×60×60 / 40×40×40
**ZrCoBi**	60×60×60 / 40×40×40
**NbCoSn**	60×60×60 / 40×40×40
**TaCoSn**	60×60×60 / 40×40×40

Supplementary Table 3. Room temperature thermal conductivities calculated in this work compared to previous simulation[18].

Material	Thermal conductivity (W/m-K) (this work)	Thermal conductivity (W/m-K) (literature)
**NbFeSb**	27.0	29.1
**ScNiSb**	26.6	19.5
**ScNiBi**	20.9	14.3
**ZrNiSn**	18.5	17.5
**ZrCoSb**	24.7	24.4

The calculated thermal conductivities for the two compounds with Sc are higher than previous simulation results, probably due to the different choices in fitting the (harmonic / anharmonic) force constants. The agreement is in general good.

Supplementary Table 4. Predicted intrinsic mobility at room temperature for half-Heusler materials compared with experimental intrinsic mobilities from various material families.

Family	Material	Experiment ($cm^2 V^{-1} s^{-1}$)	Simulation ($cm^2 V^{-1} s^{-1}$)
III-V	InP (n)	5370[51]	N/A
	InAs (n)	33000[51]	N/A
	InSb (n)	60000[51]	N/A
	GaAs (n)	9200[51]	N/A
	GaSb (n)	6000[51]	N/A
Elemental	Si (n)	1550[51]	N/A
	Ge (n)	4600[51]	N/A
	Te (p)	1260[51]	N/A
Skutterudite	$CoSb_3$ (p)	3300[52]	N/A
Chalcogenide	PbTe (n)	2000[53]	N/A
	PbTe (p)	350[54]	N/A
	PbSe (n)	1000[55]	N/A
$(Bi,Sb)_2(Te,Se)_3$	Bi_2Te_3 (n)	1440[51]	N/A
	Bi_2Se_3 (n)	780[51]	N/A
	Sb_2Te_3 (p)	270[51]	N/A
Half-Heusler	NbFeSb	27 (p)[25]	500 (n) / 77 (p)
	ScNiSb	N/A	180 (n) / 240 (p)
	YNiSb	N/A	150 (n) / 330 (p)
	ScNiBi	N/A	180 (n) / 380 (p)
	YNiBi	N/A	210 (n) / 690 (p)
	TiNiSn	30 (n)[56]	200 (n) / 56 (p)
	ZrNiSn	70 (n)[56]	300 (n) / 53 (p)
	HfNiSn	N/A	390 (n) / 78 (p)
	ZrNiPb	60 (n)[57]	290 (n) / 350 (p)

	TiCoSb	N/A	32 (n) / 30 (p)
	ZrCoSb	8 (p)[58]	54 (n) / 45 (p)
	HfCoSb	N/A	70 (n) / 40 (p)
	ZrCoBi	N/A	50 (n) / 46 (p)
	NbCoSn	N/A	64 (n) / 47 (p)
	TaCoSn	N/A	92 (n) / 60 (p)

The carrier type is indicated inside the bracket. The density-of-state effective mass are taken from the references listed and normalized to each valley based on their valley degeneracy at the corresponding band edge. The calculated mobilities presented here include the following effects as mentioned in text: polar scattering, screening effect due to free carriers and electron scattering by ionized impurities.

Supplementary Table 5. Predicted optimal power factors at room temperature as well as the corresponding carrier concentrations for half-Heusler compounds.

**Power factor [μW cm^{-1} K^{-2}] (optimal carrier density [cm^{-3}])**	**n-type**	**p-type**
**NbFeSb**	92 (1×10^{20})	72 (2×10^{20})
**ScNiSb**	90 (1×10^{20})	105 (1×10^{20})
**YNiSb**	84 (1×10^{20})	72 (4×10^{19})
**ScNiBi**	100 (1×10^{20})	126 (6×10^{19})
**YNiBi**	100 (1×10^{20})	89 (4×10^{19})
**TiNiSn**	83 (1×10^{20})	78 (4×10^{20})
**ZrNiSn**	89 (1×10^{20})	58 (2×10^{20})
**HfNiSn**	120 (1×10^{20})	72 (2×10^{20})
**ZrNiPb**	106 (1×10^{20})	113 (6×10^{19})
**TiCoSb**	48 (4×10^{20})	49 (4×10^{20})
**ZrCoSb**	59 (2×10^{20})	53 (2×10^{20})
**HfCoSb**	72 (2×10^{20})	53 (2×10^{20})
**ZrCoBi**	66 (4×10^{20})	57 (2×10^{20})
**NbCoSn**	59 (2×10^{20})	58 (2×10^{20})
**TaCoSn**	71 (2×10^{20})	76 (2×10^{20})

The power factors obtained here include the following effects as mentioned in text: polar scattering, screening effect due to free carriers and electron scattering by ionized impurities.

Supplementary Table 6. Measured power factors taken at their maximum points for series of half-Heusler compounds.

Power factors [$\mu W\ cm^{-1}\ K^{-2}$]	n-type	p-type
**NbFeSb**	N/A	106[25], $Nb_{0.95}Ti_{0.05}FeSb$
**TiCoSb**	N/A	26[59], $TiCoSb_{0.8}Sn_{0.2}$
**ZrCoSb**	33[60], $ZrCo_{0.9}Ni_{0.1}Sb$	30[58], $ZrCoSb_{0.85}Sn_{0.15}$
**HfCoSb**	N/A	35[59], $HfCoSb_{0.8}Sn_{0.2}$
**ZrCoBi**	8[61], $ZrCo_{0.95}Ni_{0.05}Bi$<br>29*, $ZrCo_{0.9}Ni_{0.1}Bi$	N/A<br>39*, $ZrCoBi_{0.8}Sn_{0.2}$
**NbCoSn**	35[62], $NbCoSn_{0.9}Sb_{0.1}$	N/A
**TaCoSn**	N/A	N/A
**ScNiSb**	4.2[63]<br>$Sc_{0.98}Ti_{0.02}Ni_{0.98}Cu_{0.02}Sb_{0.98}Te_{0.02}$	6.7[63]<br>$ScNi_{0.98}Co_{0.02}Sb_{0.98}Sn_{0.02}$
**YNiSb**	N/A	N/A
**ScNiBi**	N/A	N/A
**YNiBi**	N/A	13.3[64], YNiBi
**TiNiSn**	40[56], $Nb_{0.02}Ti_{0.98}NiSn$	N/A
**ZrNiSn**	52[21], $ZrNiSn_{0.99}Sb_{0.01}$	N/A
**HfNiSn**	38[65], HfNiSn	N/A
**ZrNiPb**	54[57], $ZrNiPb_{0.993}Bi_{0.007}$	N/A

The listed power factors represent the highest values for each composition reported so far. Compositions with alloying on atomic sites (e.g. $Zr_{0.5}Hf_{0.5}NiSn$) are not considered in this list because their band structure may deviate significantly from that of single crystal considered in this work. The detailed compositions that achieve such power factor values are given in the table. The values with references are from literature, while those labeled with asterisk (*) are obtained in this work. Note that ScNiSb and YNiBi exhibit very small experimental power factors because these samples are not well optimized with respect to their carrier density.

Supplementary Table 7. Predicted optimal power factors at 1000K as well as the corresponding carrier concentrations for select half-Heusler compounds.

Material	Optimal carrier density (cm^{-3})	Optimal power factor ($\mu W\ cm^{-1}\ K^{-2}$)
**n-type NbFeSb**	~2e20	70
**p-type ScNiBi**	~4e20	70
**n-type ScNiSb**	~6e20	55
**p-type ScNiSb**	~4e20	71
**p-type ZrNiPb**	~6e20	42

Supplementary Table 8. Comparison of total energies of electronic structure calculations with and without initialization of magnetizations for NbFeSb and ZrNiSn.

Total energy (Rydberg)	With initial magnetization	Without magnetization
**NbFeSb**	-825.23345827	-825.23345829
**ZrNiSn**	-936.46269776	-936.46269776

The starting condition for magnetization before electronic calculations assumes 1.0 on Fe (Ni) atom, and the final results converge to zero magnetization. The results are obtained using fully-relativistic PAW pseudopotentials[66] with spin-orbit coupling.

Supplementary Table 9. Character table of D_{2d} point group.

	E	$2S_4$	$C_2(z)$	$2C'_2$	$2\sigma_d$	linear	Quadratic
A_1	1	1	1	1	1		$x^2+y^2+z^2$, z^2
A_2	1	1	1	-1	-1		
B_1	1	-1	1	1	-1		x^2-y^2
B_2	1	-1	1	-1	1	z	xy
E	2	0	-2	0	0	x, y	xz, yz

Supplementary Table 10. Character table of C_{3v} point group.

	E	$2C_3(z)$	$3\sigma_v$	linear	Quadratic
A_1	1	1	1	z	$x^2+y^2+z^2$, z^2
A_2	1	1	-1		
E	2	-1	0	x, y	x^2-y^2, xy, xz, yz

Supplementary Table 11. Character table of T_d point group.

	E	$8C_3$	$3C_2$	$6S_4$	$6\sigma_d$	linear	Quadratic
A_1	1	1	1	1	1		$x^2+y^2+z^2$
A_2	1	1	1	-1	-1		
E	2	-1	2	0	0		x^2-y^2, z^2
T_1	3	0	-1	1	-1		
T_2	3	0	-1	-1	1	x, y, z	xy, xz, yz

Supplementary Note 1. Thermoelectric transport calculation

Electron scattering rates are calculated for each electron state by summing over all possible scattering channels based on a tetrahedral integration method[1]. Combining them with other quantities (e.g. group velocities) that are readily obtained from band structure information, one can then calculate the transport properties such as electrical conductivity and Seebeck coefficient based on the Boltzmann transport equation[2–4], as already discussed in the Methods section. The convergence with respect to the electron and phonon meshes has been carefully checked, and depending on the density of states different mesh densities are used for different compositions, and are listed in Supplementary Table 2. We also note that as dictated by the Fermi-Dirac distribution, only electrons within several k_BT's (~0.026 eV for room temperature) from the band edge (or Fermi level if it is inside the band) will dominate the transport. Therefore, to avoid unnecessary calculations we have only considered the electron states that are within 0.3 eV from the band edge, which has been checked to be enough for room temperature calculations. For higher temperature, this value is increased to ensure that the electron states with major contributions to the transport have been included in the calculation.

We particularly mention that the interpolation scheme using Wannier functions (EPW)[5,6] needs to be carefully checked, especially for the band structure and the electron-phonon coupling matrix. In general, including more atomic orbitals leads to better fitting of the original band structure from first principles, but also makes the electron scattering rate calculation more computationally heavy. As a balance, we found a number of 19 atomic orbitals leads to well-interpolated band structure, with *s* and *d* orbitals on A, *s*, *p* and *d* orbitals on B, as well as *s* and *p* orbitals on C. The frozen window[7] is taken to include all of the valence band, and the number of bands as input from first principles is 27. In Supplementary Fig. 11a we show a comparison between the interpolated band structure and that originally obtained from first principles calculation for ZrNiSn. We have checked other compositions and they all show similar agreement. Smaller number of atomic orbitals however cannot interpolate the band structure well.

Regarding the electron-phonon coupling (EPC) matrix, we found that the interpolation does not work very well for the acoustic phonon branch in the half-Heusler material (for simpler III-V semiconductor with no *d* atomic orbitals involved, the interpolation is reasonably good). Particularly, at high symmetry lines where the coupling matrix associated with certain modes (e.g.

transverse modes) should vanish, the interpolated EPI gives non-zero results. Therefore in obtaining the EPI and deformation potentials as shown in Fig. 1, we have used results directly obtained from the first principles calculation, without any interpolation. However, we note that the order of magnitude of the interpolated EPC is similar to the first principles result, with the difference being usually less than 30%. On the other hand, the interpolation for the optical phonon branch is generally good, as shown in Supplementary Fig. 11b. Because in half-Heuslers the charge transport is dominated by the optical phonon scattering, the calculated transport properties do not suffer much from the less accurately interpolated electron-acoustic phonon coupling matrices in EPW. We have further checked this by linearly interpolating the EPC to fine meshes and perform transport calculations. The results for several materials show less than 10% variation compared with the values we report in the paper obtained via EPW interpolation, thereby justifying the validity of our simulation procedure. We further note that the electron transport calculations employing the EPW interpolation for EPI's has led to several successful demonstrations recently in a series of benchmarked materials, including Si[8,9], GaAs[10–12], perovskites[13] and two-dimensional materials[9,14,15].

For the phonon mean free path and thermal conductivity calculation, we used our home-built code as described in previous publications[16,17]. The harmonic force constants are fitted up to 5th nearest neighbor shell while the 3rd order anharmonic force constants are fitted up to the second nearest neighbor shell[16]. They are used to calculate the phonon scattering rates, which then give the phonon transport properties[17], where a phonon mesh of $30 \times 30 \times 30$ is found to reach the convergence. In Supplementary Table 3 we compare the obtained thermal conductivity values with a recent high throughput calculation[18] on half-Heusler compounds at room temperature. The agreement is generally very good.

In order to have a realistic evaluation of the thermoelectric performance, we consider practical effects that also influence the electron transport, including the polar scattering, the screening effect on the polar scattering and the ionized impurity scattering due to the dopants. We regard such calculated power factors as the intrinsic power factors, because practical materials may have defects which could introduce extrinsic impurity scatterings that are not included in this work. The first two effects will be dealt with in Supplementary Note 2. Here we briefly mention

our treatment for the electron-impurity scattering. The electron impurity scattering rate can be estimated based on the transition matrix[3]:

$$H_{\mathrm{e-imp}} = \langle \psi_{\mathbf{k}} | \Delta V | \psi_{\mathbf{k}'} \rangle \tag{1}$$

where ΔV is the potential change induced by the ionized impurity. If we approximate this impurity potential by its long-range Coulomb part and the wavefunctions simply as plane waves, we obtain the following form:

$$H_{\mathrm{e-imp}} \approx \frac{e^2}{\Omega \varepsilon \varepsilon_0} \frac{1}{q^2 + 1/L_{\mathrm{D}}^2} \tag{2}$$

where Ω is the unit cell volume, ε is the material's relative dielectric constant, ε_0 is the vacuum permittivity, and q is the change of the electron wave vector. This is consistent with the assumption used to derive the Brooks-Herring model for ionized impurity scattering[3,19]. We note that the effect of carrier screening has been taken into account in the Debye length L_{D}[20]. For general band structure it can be derived that[3]

$$L_{\mathrm{D}} = \left(\frac{e^2}{\varepsilon \varepsilon_0} \int \left(-\frac{\partial f}{\partial E} \right) D(E) \mathrm{d}E \right)^{-1/2} \tag{3}$$

where $D(E)$ is the density of states. Based on the Fermi's Golden rule, one can then write down the scattering rate under the momentum relaxation time approximation[3]:

$$\frac{1}{\tau_{\mathrm{imp}}(\mathbf{k})} = N_{\mathrm{imp}} \Omega \frac{2\pi}{\hbar} \left(\frac{e^2}{\Omega \varepsilon \varepsilon_0} \right)^2 \sum_{\mathrm{m}} \int \frac{1}{(q^2 + 1/L_{\mathrm{D}}^2)^2} \left(1 - \frac{\mathbf{v_k} \cdot \mathbf{v_{k+q}}}{|\mathbf{v_k}||\mathbf{v_{k+q}}|} \right) \delta(E_{\mathbf{k}} - E_{\mathbf{k+q}}) \mathrm{d}\mathbf{q} \tag{4}$$

where the factor $\left(1 - \frac{\mathbf{v_k} \cdot \mathbf{v_{k+q}}}{|\mathbf{v_k}||\mathbf{v_{k+q}}|} \right)$ takes into account the fact that backward scatterings change the electron momentum much more than forward scatterings. The obtained impurity scattering rates are then added to the electron-phonon scattering rates according to the Matthiessen's rule:

$$\frac{1}{\tau_{\mathrm{tot}}} = \frac{1}{\tau_{\mathrm{e-ph}}} + \frac{1}{\tau_{\mathrm{imp}}} \tag{5}$$

With the electron scattering rates, one can calculate the transport properties. In the main text we have shown the comparison for the electrical transport properties (conductivity, Seebeck coefficient and power factor) between simulation and experiment in p-type NbFeSb. The good agreement across a wide temperature range justified our simulation framework. Here we want to

show such comparison in another well-known half-Heusler thermoelectric material – n-type ZrNiSn, to illustrate an important point made in our manuscript, about the discrepancy between simulation and experiment. The electron mobility (defined as $\mu = \frac{\sigma}{ne}$ where n is the carrier density) and Seebeck coefficient as functions of carrier concentrations are shown in Supplementary Fig. 12 for ZrNiSn. For this we have included all practical effects as mentioned in sections above. The Seebeck coefficient does not sensitively depend on whether we consider impurity scattering or not (mainly depends on the density of states near the band edge). Despite the fact that the predicted Seebeck coefficient is slightly smaller, the trend agrees well with the experiments[21–24]. The small Seebeck coefficient might be due to the underestimation of the density-of-states effective mass in ZrNiSn. The experimental data can be well characterized by a density-of-states effective mass of $\sim 3.0 m_e$ assuming parabolic band (as shown by the dashed line in Supplementary Fig. 12), while our calculation corresponds to an effective mass of $\sim 2.0 m_e$. Such discrepancy is understandable within first principles as it is well-known the band gap and sometimes the band shape will deviate from the measured values. Nonetheless this will not change our qualitative understanding of the electron transport. A more distinct discrepancy between simulation and experiment is found for the mobility. The calculated mobility is significantly larger than the experimental values, which cannot be attributed to the first principles method. Though short-range impurity scatterings that are not included in our modeling may affect the electron transport, recent work by Xie *et al*[21] explains that excess Ni creates significant alloy scattering that will severely limit the charge transport. We have found similar results for other half-Heusler compounds – the Seebeck coefficient generally matches experiments but the predicted mobility is higher, explaining the relatively high power factors compared to experiments as shown in Fig. 4a. As we have mentioned in the main text, the high power factor predicted from first principles in general hints at the fact that there are still significant amounts of defects in practical materials. Therefore if we can reduce the defect concentrations one would expect higher mobility and also larger power factors in practical half-Heusler materials.

To determine the optimal power factor we have performed the calculation for a range of carrier concentrations. The optimal carrier concentration reaching these and the corresponding optimal power factors are given in Supplementary Table 4.

We also want to briefly mention the temperature dependence of the transport properties, which is highly relevant for experimental studies. First, it is a common practice to judge whether the charge transport is consistent with the acoustic phonon scattering model by examining the temperature scaling of the electron mobility μ[25,26]. It is known that acoustic phonon dominated transport yields $\mu \propto T^{-3/2}$[26]. The experimentally measured mobility indeed often varies with temperature like this, thereby giving us the "confidence" that the electrons are mainly scattered by acoustic phonons. However, we want to note that optical phonon dominated transport can also lead to similar temperature scaling. In Supplementary Fig. 13 we illustrate this point by calculating the mobility assuming either acoustic phonon scattering or optical phonon scattering (non-polar) based on the simple parabolic band model in the non-degenerate regime. By fitting the curve at high temperatures (>500K) we found for the acoustic phonon case, $\mu \propto T^{-1.5}$ while for the optical phonon case $\mu \propto T^{-1.7}$. These temperature scaling ratios are within experimental errors. This questions the common practice that directly relates the temperature scaling to specific phonon branch in scattering electrons, let alone the fact that in practice the temperature scaling for acoustic phonons can deviate from -0.5 due to the deviation of band structure from parabolic shape, especially when the material is in the degenerate regime. We think the experimentally observed temperature dependence of mobilities for half-Heusler materials can be equally well described by the optical phonon dominated transport (more discussions on this are in Supplementary Note 6).

The other point we want to address is the temperature dependence of the power factor. Our calculation is performed at room temperature, while most experiments report maximal power factors at high temperatures (these are also the values we compare to). The reason that their room temperature values are usually much lower is because at room temperature the thermally distributed electrons are closer to the band edge with small velocities, and thus suffer more from any present impurity scattering (typically much stronger for slower electrons). Therefore, power factors obtained at higher temperatures more closely represent the intrinsic behaviors, as is also signified by the temperature dependence of mobility (decreasing with temperature). In Supplementary Fig. 14 we again assume single parabolic band but now include both electron acoustic phonon scattering and the impurity scattering to calculate the power factor (the case for optical phonon scattering will be similar). As we can see, the intrinsic optimal power factors (only considering acoustic phonon scattering) slightly decrease with temperature, while after adding a strong scattering term due to impurities, the power factor shows a maximal value at a high

temperature. Even if the measured power factor at higher temperatures reaches closer to the intrinsic limit, the difference in the intrinsic power factor between room temperature and high temperature may be another contributive factor of the difference we see in Fig. 4a between predicted and measured values. We note that the temperature dependence of band structures (for example, band gap, effective mass, etc) may also affect such comparison. However, the band gaps of the half-Heusler materials are generally not small (> ~0.5 eV) and the band structure variation as temperature is not very significant compared to other small band gap thermoelectric materials such as PbTe. We therefore believe our calculation results suggest that the power factor of the half-Heusler system still has much room for improvement.

Supplementary Note 2. Polar interaction for electron-phonon coupling

In this note we describe how the polar interaction is treated, and also how we add the effect of carrier screening.

The key quantity that goes into the transport calculation is the electron-phonon coupling (EPC) matrix as already given in the text[27]:

$$M_{\mathbf{k},\mathbf{q}} = \langle \psi_{\mathbf{k}} | \Delta_{\mathbf{q}} V | \psi_{\mathbf{k}+\mathbf{q}} \rangle \tag{6}$$

The perturbed potential due to the atomic displacement (corresponding to certain phonon mode) generally contains several different terms under the first principles framework[28]:

$$\Delta V(\mathbf{r}) = \Delta V_{\text{ion}}(\mathbf{r}) + e^2 \int \frac{\Delta n(\mathbf{r}')}{|\mathbf{r}-\mathbf{r}'|} d\mathbf{r}' + \left. \frac{dv_{\text{xc}}(n)}{dn} \right|_{n(\mathbf{r})} \Delta n(\mathbf{r}) \tag{7}$$

The first term is the ionic potential term (electron-ion interaction), the second term is called the Hartree term (electron-electron interaction), while the last term effectively includes all the remaining interaction that originates from the exchange correlational energy[28]. Among these, the Hartree term and the ionic potential term can give rise to long-range Coulombic forces. The long-range interactions are naturally included in the perturbed potential calculated from first principles. However, they will be lost during the interpolation process because Wannier functions are short-range functions, and the consideration of Wannier functions up to a certain distance essentially cuts off the long range interaction. In order to capture the long range interaction, one needs to first subtract the long range parts from the first principles EPC, perform the interpolation, and then add them back. This subtraction-and-addition has been based on a theoretical model that describes the effective interaction between the electrons and ions (dipole field generated by effective charges that act on the electrons), in the polar Wannier interpolation scheme[10,29]:

$$H_{\text{e-ph,polar}} = -\mathrm{i} \frac{4\pi}{\Omega} \frac{e^2}{4\pi\varepsilon\varepsilon_0} \sum_{\mathbf{G}} \frac{(\mathbf{G}+\mathbf{q}) \cdot \bar{\bar{Z}}^{\kappa} \cdot \mathbf{e}_{\kappa\lambda}(\mathbf{q})}{|\mathbf{G}+\mathbf{q}|^2} \tag{8}$$

As we are interested in the heavily-doped regime of semiconductors, the polar scattering should be largely screened by the free carriers. If we want to examine the case where polar scattering does not happen (denoted as "non-polar" case), a straightforward way is not to add the term which describes the long range interaction between electrons and ions in the EPC. In practice, we have found by neglecting the original terms in the first principles EPC that have long-range

Coulombic origin (specifically, the contributions to the Hartree and ionic potential terms that correspond to Fourier component of $\mathbf{G} = 0$[28]) and then interpolate them based on the Wannier functions, one can well describe the non-polar transport. In Supplementary Fig. 15 we show an example of the EPC associated with the optical phonon in ZrNiSn. The EPC considering the polar effect exhibits the divergence behavior $(1/q)$, characteristic of the long-range interactions, while after removing the polar interaction the EPC goes to zero at the long wavelength limit, similar as shown in Fig. 1b.

When comparing with experiments, we want to include the polar scattering together with the associated screening effect. The inclusion of the screened polar scattering generally only changes the optimal power factors for most materials by less than 20%, but nonetheless presents a more practical estimation. The polar interaction is taken into account if we add Supplementary Equation 8 back into the EPC after the interpolation. In order to effectively consider the carrier screening effect, we note that Supplementary Equation 8 is derived based on the assumption that the electrons feel the unscreened Coulombic field generated by the effective charge[30]: $\varphi(\mathbf{d}) = \frac{e}{4\pi\varepsilon d}$, with its Fourier transform $\varphi(\mathbf{q}) = \frac{e}{\varepsilon q^2}$ giving rise to the divergence seen in Supplementary Equation 8. When free carriers are present, they will screen the Coulombic field leading to a screened dipole interaction[3]: $\varphi(\mathbf{d}) = \frac{e}{4\pi\varepsilon d}\mathrm{e}^{-d/L_\mathrm{D}}$, where the Debye screening length L_D has been defined in Supplementary Note 1. The Fourier transform of this screened dipole field leads to $\varphi(\mathbf{q}) = \frac{e}{\varepsilon(q^2+1/L_\mathrm{D}^2)}$. By examining Supplementary Equation 8, we recognize that the polar EPC term considering the screening effect can be approximated as

$$H_{\mathrm{e-ph,polar}} = -\mathrm{i}\frac{4\pi}{\Omega}\frac{e^2}{4\pi\varepsilon\varepsilon_0}\sum_{\mathbf{G}}\frac{(\mathbf{G}+\mathbf{q})\cdot\bar{\bar{Z}}^{\kappa}\cdot\mathbf{e}_{\kappa\lambda}(\mathbf{q})}{|\mathbf{G}+\mathbf{q}|^2+1/L_\mathrm{D}^2} \quad (9)$$

This is the form we used to add the polar interaction into the EPC, with the screening effect contained in the Debye screening length, calculated based on the first principles band structures at different carrier concentrations as shown by Supplementary Equation 3.

Supplementary Note 3. Tight-binding analysis based on three-level system

In this note, we explain the use of a simplified tight-binding model based on a three-level system, in order to illustrate the idea of a weakly bonding or anti-bonding state, and its connection to small deformation potentials. For simplicity, we consider three levels, each of which comes from one atom with different orbital energies, as shown in Supplementary Fig. 4. To illustrate the effect, we only consider orbital interactions between the middle state with the upper and lower ones, with corresponding interaction energies labelled as V_{12} and V_{23} (real and negative numbers). With such, the Hamiltonian that determines the electronic energies with the interactions is given by

$$H = \begin{bmatrix} E_1 & V_{12} & 0 \\ V_{12} & E_2 & V_{23} \\ 0 & V_{23} & E_3 \end{bmatrix} \tag{10}$$

To give a quantitative evaluation, we put numbers into Supplementary Equation 10. For this, we have chosen E_1 = -3.5 eV, E_2 = -5 eV, E_3 = -7 eV, V_{12} = -1 eV, and V_{23} = -1.5 eV. These energy levels are chosen based on Harrison's tabulated atomic orbital energies as well as orbital interactions[31], for ZrNiSn (1: Zr, 2: Ni, 3: Sn). We have labeled the energy values on the corresponding orbital lines (Supplementary Fig. 4a).

We first focus on the middle state, which can be seen as a representative example of the valence band edge state in NbFeSb (Fig. 2f). This state's interaction with upper level (state E_1) will push itself downwards in energy, endowing it with certain bonding character (Supplementary Fig. 4b; to illustrate this, imagine only V_{12} is turned on). On the other hand, the interaction with the lower level will give it an anti-bonding character (Supplementary Fig. 4c). Now if we turn on both interactions, we will see that as a result of both bonding and anti-bonding interactions, we expect the energy of the middle level to reside in between those values shown in Supplementary Fig. 4b-c, thereby closer to the initial orbital energy E_2 (compare the middle level energy shift from -5 eV in (d) with those in (b) and (c)). That is to say, the bonding and anti-bonding interactions cancel each other, in the sense that the middle level energy is barely affected by the existence of the interactions. Such weak dependence on orbital interaction is favorable for low deformation potential, because the main source of deformation potential comes from the changes of orbital interaction energies (V_{ij}) when atoms are farther apart. If the energy level already does not depend

on V_{ij} much, the expansion of lattice then won't have big effect, translating to low deformation potentials.

To show more clearly the connection between the bonding character and the deformation potentials, we need to quantify these two things. To calculate deformation potential, we expand the lattice uniformly. According to Harrison[31], *d-d* orbital interaction (V_{23}) varies with atomic distance approximately as $\frac{1}{d^5}$, while *d-p* orbital interaction (V_{12}) varies as $\frac{1}{d^{3.5}}$. With this, one can calculate the energies for two cell sizes and subtract to obtain deformation potentials.

To quantify the bonding character, one can look at how much it deviates from its initial orbital energy. In the Supplementary Fig. 4e, we plot the deformation potentials with respect to the energy deviation from its corresponding initial atomic orbital, for all three levels. A significant positive (negative) energy shift would mean a strong anti-bonding (bonding) character, which will usually lead to large deformation potentials. This is seen for the upper and lower levels. For the middle level, because its interactions with upper and lower levels relatively cancel, its energy shift is close to zero, thereby leading to a small deformation potential. This quantitative tight-binding model illustrates the idea of the cancellation of bonding / anti-bonding interactions. Combined with the fact the valence band edge state predominantly carries *d* orbitals on atom Fe, this model indicates that this band edge state emerging from all these interactions should closely resemble a single crystal orbital, and therefore should have small deformation potentials.

In the case of ZrNiSn, we note that the CBM state corresponds to the upper level formed via orbital interactions within a three-level subset characterized by the representation B_2 (Fig. 2e). In our original drawing, this level resides above the non-bonding B_1 state, and should not be the band edge state. Meanwhile, it will also have large deformation potential as shown in Supplementary Fig. 4e. The reason it falls below B_1 state is that there must be additional interactions between B_2 and atomic orbitals at even higher energies (that are not included in our diagram), which push B_2 level lower in energy and make it smaller than B_1. To illustrate this point, in the above three-level system we introduce another energy level (E_0), and for simplicity let it only interact with the initial upper level E_1 state (the corresponding interaction denoted as V_{01}). In this case, the system becomes a four-level system, with Hamiltonian given by

$$H = \begin{bmatrix} E_0 & V_{01} & 0 & 0 \\ V_{01} & E_1 & V_{12} & 0 \\ 0 & V_{12} & E_2 & V_{23} \\ 0 & 0 & V_{23} & E_3 \end{bmatrix} \tag{11}$$

We define E_0 = -1 eV, and V_{01} = -2 eV. Solving this system with and without the V_{01}, we obtain an evolution of the energy levels as a result of this additional interaction, as shown in Supplementary Fig. 5. Without this additional interaction (Supplementary Fig. 5b), the upper level B_2 state has energy -2.87 eV and is above its initial orbital energy (-3.5 eV) and thus B_1 state. When the interaction with a higher level is turned on (Supplementary Fig. 5c), we see that its energy is pushed lower, now below the initial orbital energy (-3.5 eV). This lowering of energy is mainly a consequence of this state acquiring more bonding character due to its interactions with higher energy levels. The result of this is again a cancellation of bonding and its initial anti-bonding character, as clearly seen by its small energy shift (-3.62 eV) from the E_1 orbital energy. With this picture in mind, the observation that B_2 state lies below B_1 state then implies that now B_2 state, the conduction band edge state in ZrNiSn, should also have small deformation potential.

Supplementary Note 4. Crystal orbital group symmetry analysis

In tight-binding analysis (or linear combination of atomic orbitals, LCAO), one writes the crystal wavefunction as superpositions of atomic orbitals[32]:

$$\psi = \sum_{\mathrm{i\alpha}} c_{\mathrm{i\alpha}} \varphi_{\mathrm{i\alpha}} \tag{12}$$

where φ represents the atomic orbital of different type (ϕ) summed into its Bloch form (in our study, the 'atomic orbital' is understood as in its Bloch summation form. Because we only consider nearest neighbor interactions, the atomic orbitals in the summation do not interact with each other) and the indices i and α sum over different atomic sites and orbital types.

$$\varphi_{\mathrm{i\alpha}}(\mathbf{r}) = \frac{1}{\sqrt{N}} \sum_{\mathbf{R}_\mathrm{i}} \mathrm{e}^{\mathbf{ik\cdot R}_\mathrm{i}} \phi_{\mathrm{i\alpha}}(\mathbf{r} - \mathbf{R}_\mathrm{i}) \tag{13}$$

Knowing the band structure is equivalent to solving the eigenvalue problem derived from Schrödinger equation, defined as $\hat{H}\psi = E\psi$, where E is the electron energy. Applying the variational principle to this equation leads to a matrix equation, with prefactors $c_{\mathrm{i\alpha}}$ forming the eigenvector to be solved:

$$\mathbf{Hc} = E\mathbf{c} \tag{14}$$

The Hamiltonian matrix elements are given by $H_{\mathrm{ij}} = \langle \varphi_\mathrm{i} | \hat{H} | \varphi_\mathrm{j} \rangle$. The electron energies at a general wave vector **k** are obtained by diagonalizing the matrix *H*. Such diagonalization is often complicated and prohibits simple visualization as how the orbitals interact with each other. At a few high symmetry points for **k**, however, the group theory guarantees that certain coupling matrices are zero, meaning that for a given atomic orbital one only needs to consider its interactions with a few others[33]. This is because orbitals can be categorized by the group theory into different so-called irreducible representations, and those belonging to different representations do not interact with each other[34]. To illustrate how this works, we take half-Heusler materials (denoted as ABC, like in ZrNiSn) with **k** at the X point as an example.

For the half-Heusler materials, we choose a minimal basis set, where *d* orbitals for atoms A and B, as well as *s* and *p* orbitals for atom C are considered (in total 14 atomic orbitals, so we need to solve 14-by-14 matrix in Supplementary Equation 14). These are listed below:

$$\varphi_{\mathrm{A},d_{\mathrm{z}^2}}, \varphi_{\mathrm{A},d_{\mathrm{x}^2-\mathrm{y}^2}}, \varphi_{\mathrm{A},d_{\mathrm{xy}}}, \varphi_{\mathrm{A},d_{\mathrm{yz}}}, \varphi_{\mathrm{A},d_{\mathrm{xz}}},$$

$$\varphi_{\mathrm{B},d_{z^2}}, \varphi_{\mathrm{B},d_{x^2-y^2}}, \varphi_{\mathrm{B},d_{xy}}, \varphi_{\mathrm{B},d_{yz}}, \varphi_{\mathrm{B},d_{xz}},$$

$$\varphi_{\mathrm{C},s}, \varphi_{\mathrm{C},p_x}, \varphi_{\mathrm{C},p_y}, \varphi_{\mathrm{C},p_z} \tag{15}$$

At X-point, the symmetry group corresponding to the Hamiltonian is D_{2d} (also called small group of **k**)[35], which is reduced from the crystal symmetry group (T_d), because only the symmetry operations within this small group do not alter the wave vector **k**. To characterize the atomic orbitals (in the form of Bloch sum) as given by Supplementary Equation 15, we note that the result of a point symmetry transformation of φ_i is actually the product of two transformations[34]: One is the transformation of the sub-lattice phase factor $P_{\mathbf{k}}^{i} = \sum_{\mathbf{R}_i} e^{i\mathbf{k}\cdot\mathbf{R}_i}$ and the other is the transformation of the atomic orbital ϕ_i on a single atomic site. As a result, we need to figure out how each of them transforms under the symmetry operations (their representations).

The representation of the single-site atomic orbitals (ϕ_i) corresponding to D_{2d} can be easily found by checking the character table of D_{2d}, noting that p_x transforms like x function, d_{xy} transforms like xy function and s transforms as $x^2+y^2+z^2$. We should mention such functions are defined relative to the coordinate system, and in our case we take the x, y, z axis to be parallel with the edge of the cubic cell ($\mathbf{k}_X$ along z-direction), and the sub-lattice positions to be A = (0, 0, 0), B = (1/4, 1/4, 1/4) and C = (1/2, 1/2, 1/2), in units of lattice constant. The character table, in brief word, is a two-dimensional description of different representations and how each of them transforms under symmetry operations[34]. The rows denote different irreducible representations, and the columns correspond to inequivalent groups of symmetry operations. The entries are called characters, which allow algebraic operations when considering representation of a product function. The character table of D_{2d} is given in Supplementary Table 9[36].

The analysis for the single-site atomic orbitals leads to the following categorization of the 9 different orbital types:

A_1: s
B_1: d_{x2-y2}
B_2: p_z, d_{xy}
E: p_x, p_y, d_{xz}, d_{yz}

On the other hand, the categorization of the sub-lattice phase factor $P_{\mathbf{k}}^{i} = \sum_{\mathbf{R}_i} e^{i\mathbf{k}\cdot\mathbf{R}_i}$ is found by following routine group theory analysis[34] – check how this function transforms under symmetry

operations. There are three such different phase factors, corresponding to three different sub-lattice points (denoted as $P_{\mathrm{A}}^{\mathrm{i}}$, $P_{\mathrm{B}}^{\mathrm{i}}$ and $P_{\mathrm{C}}^{\mathrm{i}}$). Take B as an example, the symmetry operation S_4 will flip the sign and therefore is equivalent as a constant factor of -1 in the one-dimensional representation. By examining through all the symmetry operations and comparing the results with the character table, one can identify that $P_{\mathrm{B}}^{\mathrm{i}}$ belongs to the B_2 representation. Repeating this analysis leads to the following categorization:

A_1: $P_{\mathrm{A}}^{\mathrm{i}}, P_{\mathrm{C}}^{\mathrm{i}}$
B_2: $P_{\mathrm{B}}^{\mathrm{i}}$

The representation of the atomic orbitals (Supplementary Equation 15) is a direct product of two representations. To find out the representation, based on the group theory for the presentation of a product function[34], one take the characters of each of the two representations, multiply them together, and check which representation the product now corresponds to. In doing so, we finally obtain the symmetry categorization for all the 14 orbitals needed in the tight binding analysis:

A_1: $\varphi_{\mathrm{A},d_{\mathrm{z}^2}}, \varphi_{\mathrm{B},d_{\mathrm{xy}}}, \varphi_{\mathrm{C},s}$
A_2: $\varphi_{\mathrm{B},d_{\mathrm{x}^2-\mathrm{y}^2}}$
B_1: $\varphi_{\mathrm{A},d_{\mathrm{x}^2-\mathrm{y}^2}}$
B_2: $\varphi_{\mathrm{A},d_{\mathrm{xy}}}, \varphi_{\mathrm{B},d_{\mathrm{z}^2}}, \varphi_{\mathrm{C},p_{\mathrm{z}}}$
E: $\varphi_{\mathrm{A},d_{\mathrm{xz}}}, \varphi_{\mathrm{A},d_{\mathrm{yz}}}, \varphi_{\mathrm{B},d_{\mathrm{xz}}}, \varphi_{\mathrm{B},d_{\mathrm{yz}}}, \varphi_{\mathrm{C},p_{\mathrm{x}}}, \varphi_{\mathrm{C},p_{\mathrm{y}}}$

When forming the orbital interaction diagram (Fig. 2e), we also need to decide where the atomic orbitals (before interaction) are approximately located[37]. This can be estimated by the atomic orbital energy levels. Though such determination of energy levels is not accurate, it will not affect the qualitative picture of orbital bonding (for example where the bonding, anti-bonding and non-bonding orbitals are). The advantage of the symmetry analysis, is to reduce the number of interactions we have to consider when constructing such orbital bonding pictures. For example, it is clear that the orbital $\varphi_{\mathrm{A},d_{\mathrm{x}^2-\mathrm{y}^2}}$ does not interact with nearby orbitals (in the context that only nearest neighbor interactions are considered between A-B, A-C and B-C pairs), thereby representing a distinct non-bonding state. On the other hand, for representation E, only orbitals among $\varphi_{\mathrm{A},d_{\mathrm{xz}}}, \varphi_{\mathrm{A},d_{\mathrm{yz}}}, \varphi_{\mathrm{B},d_{\mathrm{xz}}}, \varphi_{\mathrm{B},d_{\mathrm{yz}}}, \varphi_{\mathrm{C},p_{\mathrm{x}}}, \varphi_{\mathrm{C},p_{\mathrm{y}}}$ will interact. Because E is a two-dimensional representation, the interaction of these six orbitals will form three doubly-degenerate states[34], with one bonding state at lower energy, one anti-bonding state at higher energy, and one with energy in

between. The state in the middle often has minimal bonding or anti-bonding interactions, due to the cancellation facilitated by its interactions with both higher and lower-energy orbitals[37]. With such proper energetic interactions, the charge density of this state can be well localized at a single atomic state (its energy level will also be close to the atomic orbital energy). In this regard, one often refers to this state similarly as non-bonding state[38]. We should note that although its bonding and anti-bonding interactions do not exactly cancel as in representation B_1, the associated vanishing bonding or anti-bonding interactions are still protected by the symmetry, because the latter prohibits this state's interactions with other orbitals that would otherwise introduce significant bonding / anti-bonding interactions. The orbitals for the valence band edge (with one example from Fig. 2f) belong to this class.

Having gone through the symmetry analysis at X-point, similar analysis can be done at other high symmetry points. The conduction band edges mostly occur at X in half-Heuslers, while the valence band edges occur both at L and Γ. Without repeating the detailed derivation as elaborated above, we list below the character table of their symmetry groups and the symmetry categorization of the atomic orbitals[36].

At L-point, we make the coordinate system so that $\mathbf{k}_L$ parallels with z-direction. The small group at L-point is C_{3v}, and its corresponding character table is given in Supplementary Table 10.

The categorization of the 14 atomic orbitals (Supplementary Equation 15) leads to:

A_1: $\varphi_{\mathrm{A},d_{\mathrm{z}^2}}, \varphi_{\mathrm{B},d_{\mathrm{z}^2}}, \varphi_{\mathrm{C},p_{\mathrm{z}}}, \varphi_{\mathrm{C},s}$

E: $\varphi_{\mathrm{A},d_{\mathrm{x}^2-\mathrm{y}^2}}, \varphi_{\mathrm{A},d_{\mathrm{xy}}}, \varphi_{\mathrm{A},d_{\mathrm{yz}}}, \varphi_{\mathrm{A},d_{\mathrm{xz}}}, \varphi_{\mathrm{B},d_{\mathrm{x}^2-\mathrm{y}^2}}, \varphi_{\mathrm{B},d_{\mathrm{xy}}}, \varphi_{\mathrm{B},d_{\mathrm{yz}}}, \varphi_{\mathrm{B},d_{\mathrm{xz}}}, \varphi_{\mathrm{C},p_{\mathrm{x}}}, \varphi_{\mathrm{C},p_{\mathrm{y}}}$

The corresponding orbital interaction diagram is given in Fig. 2f.

At Γ-point, the coordinate system is chosen with x, y, z axis parallel with the edges of the cubic cell. Now the group symmetry is the full crystal symmetry T_d, and the corresponding character table is given in Supplementary Table 11.

The categorization of the 14 atomic orbitals (Supplementary Equation 15) leads to:

A_1: $\varphi_{\mathrm{C},s}$

E: $\varphi_{\mathrm{A},d_{\mathrm{x}^2-\mathrm{y}^2}}, \varphi_{\mathrm{A},d_{\mathrm{z}^2}}, \varphi_{\mathrm{B},d_{\mathrm{x}^2-\mathrm{y}^2}}, \varphi_{\mathrm{B},d_{\mathrm{z}^2}}$

T_2: $\varphi_{\mathrm{A},d_{\mathrm{xy}}}, \varphi_{\mathrm{A},d_{\mathrm{yz}}}, \varphi_{\mathrm{A},d_{\mathrm{xz}}}, \varphi_{\mathrm{B},d_{\mathrm{xy}}}, \varphi_{\mathrm{B},d_{\mathrm{yz}}}, \varphi_{\mathrm{B},d_{\mathrm{xz}}}, \varphi_{\mathrm{C},p_{\mathrm{x}}}, \varphi_{\mathrm{C},p_{\mathrm{y}}}, \varphi_{\mathrm{C},p_{\mathrm{z}}}$

In Supplementary Fig. 2 we have plotted the orbital interaction diagram according to this categorization. It is clear from Supplementary Fig. 2 and Fig. 2f that the valence band edge orbitals are indeed characterized by nearly non-bonding orbitals, with vanishing bonding or anti-bonding interactions, which render weak electron-acoustic phonon scatterings and therefore benefit the mobility.

Supplementary Note 5. Crystal orbital Hamilton population analysis

We defined and briefly explained the concept of crystal orbital Hamilton population in the Methods section. In this note we give a general discussion on its development and also a simple derivation that will more clearly illustrate the idea.

The whole idea originates from Mulliken's original definition for orbital overlap population given for molecules (Mulliken population analysis)[39,40]. Take the hydrogen molecule as an example, if we are to construct a molecular orbital, we will seek to combine the atomic orbitals on the two sites ($\phi_{1,2}$), giving rise to[41,42]

$$\psi = c_1\phi_1 + c_2\phi_2 \tag{16}$$

The prefactors ($c_{1,2}$) contain information that whether the interaction between atomic orbitals is bonding-like or anti-bonding-like[42]. In general when the interaction between atomic orbitals favors lower energy, one finds for this simple example that for the bonding state $c_1 = c_2$ while for the anti-bonding state $c_1 = -c_2$. If we define a quantity as $c_1^* c_2$, its positive or negative value then indicates the interaction as bonding or anti-bonding like. This is the original definition by Mulliken for quantifying the degree of bonding in the molecules[40]. However, it does not distinguish between different bond strengths[43]. For example, if we consider the molecular orbital constructed from same *p* orbital instead of the *s* orbital, the above analysis will yield the same number, while in practice we know *s*-*s* interactions should be stronger than the *p*-*p* interactions. To take bond strength effect into account, one can weight the Mulliken formula by the interaction potential (H_{12} characterizing the energetic interaction between the orbitals), leading to $c_1^* H_{12} c_2$. This form can be derived by partitioning the energy into contributions from each orbital pair, as we will show below.

The above discussion is for isolated molecules, but the concept should be generally applicable. The idea of population analysis was later generalized by Hoffman to understand solid state materials[44], and in this case, the molecular orbitals are replaced by the crystal orbitals, which are superpositions of atomic orbitals on each lattice point. The original development by Hoffman was focused on Mulliken's definition, which led to the concept of crystal orbital overlap population (COOP)[45]. A parallel development taking into account the bond strength (interaction potential) leads to the crystal orbital Hamilton population (COHP)[46], which is more useful for comparing

different materials that have more than one orbital type, and therefore is what we used in studying the bonding character. Below we give a simple derivation for the COHP.

We have the crystal orbital written as summations of atomic orbitals:

$$\psi = \sum_{i} c_{i}\varphi_{i} \tag{17}$$

where φ represents atomic orbitals in its Bloch form and the index i sums over all atomic orbitals at different atomic sites. The Schrodinger equation that governs the eigen-energies of this material leads to

$$\hat{H}\psi_{n} = \varepsilon_{n}\psi_{n} \tag{18}$$

Here φ_{i}'s are understood to be orthogonal to each other. We note that any atomic orbital basis that is not orthogonal can always be transformed into the so-called Löwdin orthogonalized basis[47]. For a given eigen-state described by ψ_{n}, the associated eigen-energy can be written as

$$\begin{aligned} \varepsilon_{n} &= \langle\psi_{n}|\hat{H}|\psi_{n}\rangle \\ &= \sum_{i,j} c_{i}^{n*} c_{j}^{n} H_{ij} \\ &= \sum_{i}|c_{i}^{n}|^{2}H_{ii} + 2\sum_{i<j}\mathrm{Re}(c_{i}^{n*}c_{j}^{n}H_{ij}) \end{aligned} \tag{19}$$

where $H_{ij} = \langle\varphi_{i}|\hat{H}|\varphi_{j}\rangle$ is the Hamiltonian matrix element. In this form, the first term represents the contributions from the on-site energies, while the second term is due to the interactions between atomic orbitals on different sites. This second term is defined as the COHP for a given state (considering all orbital pairs).

$$\mathrm{COHP}_{\mathbf{kn}} = 2\sum_{i<j}\mathrm{Re}(c_{i}^{\mathbf{kn}*}c_{j}^{\mathbf{kn}}H_{ij}) \tag{20}$$

Summing over all the states at a given energy, one obtains the COHP for the crystal as a function of energy, similar to the density of states:

$$\begin{aligned} \mathrm{COHP}(E) &= \sum_{i<j}\mathrm{COHP}_{ij}(E) \\ &= \sum_{i<j}\sum_{\mathbf{kn}} 2\mathrm{Re}(c_{i}^{\mathbf{kn}*}c_{j}^{\mathbf{kn}}H_{ij})\cdot\delta(E - E_{\mathbf{kn}}) \end{aligned} \tag{21}$$

This is often used to identify the major contributions to the bonding / anti-bonding extent[48]. However, this value is masked by the density of states and is less useful to quantitatively compare

different materials. Therefore, we have evaluated the COHP using Supplementary Equation 20 for the (conduction) band edge state, as plotted in Supplementary Fig. 3.

For calculating COHP's, the first principles wavefunctions are projected onto the Löwdin orthogonalized atomic wavefunctions, which give the coefficients (c_i 's) that appear in Supplementary Equation 20. In this regard our calculated COHP's should be strictly referred to as pCOHP ("p" means projection)[49]. COHP's for conduction band edge states are then readily calculated by looking at the crystal population analysis for the wavefunction at the band edge point.

Supplementary Note 6. Optical phonon limited charge transport

We have found in the main text that for half-Heusler materials, because the electron-acoustic phonon scattering is largely suppressed, the optical phonons become important for the charge transport. In this note we discuss the general features of the charge transport if optical phonons dominate the electron scatterings, and compare it with the acoustic phonon limited transport. For illustration we will assume single parabolic band and energy-dependent relaxation times.

The microscopic formula for the electrical conductivity and Seebeck coefficient are written as[50]

$$\sigma = \frac{e^2}{3\Omega N_{\mathbf{k}}} \sum_{\mathbf{k}n} \mathbf{v}_{\mathbf{k}n}^2 \tau_{\mathbf{k}n} \left(-\frac{\partial f_{\mathbf{k}n}}{\partial E}\right) \tag{22}$$

$$S = \frac{e}{3\sigma\Omega N_{\mathbf{k}} T} \sum_{\mathbf{k}n} (E_{\mathbf{k}n} - \mu) \mathbf{v}_{\mathbf{k}n}^2 \tau_{\mathbf{k}n} \left(-\frac{\partial f_{\mathbf{k}n}}{\partial E}\right) \tag{23}$$

For parabolic band, the velocity only depends on the electron energy. We have also assumed energy dependent electron relaxation time, essentially neglecting its dependence on the direction of $\mathbf{k}$. Therefore the above formula can be transformed into integration over the electron energy:

$$\sigma = \frac{e^2}{3} \int_0^\infty \mathbf{v}_{\mathrm{E}}^2 \tau_{\mathrm{E}} D(E) \left(-\frac{\partial f}{\partial E}\right) \mathrm{d}E \tag{24}$$

$$S = \frac{e}{3\sigma T} \int_0^\infty \mathbf{v}_{\mathrm{E}}^2 \tau_{\mathrm{E}} (E - \mu) D(E) \left(-\frac{\partial f}{\partial E}\right) \mathrm{d}E \tag{25}$$

where $D(E) = \frac{1}{2\pi^2} \left(\frac{2m^*}{\hbar^2}\right)^{3/2}$ is the density of states. For parabolic band one has $\mathbf{v}_{\mathrm{E}}^2 = \frac{2E}{m^*}$, where m^* is the effective mass. The deformation potential approximation further leads to the following results for the relaxation times, for acoustic phonon and optical phonon scattering respectively (for optical phonon we do not consider polar scattering)[3]:

$$\frac{1}{\tau_{\mathrm{E,ac}}} = \frac{\pi D_{\mathrm{A}}^2 k_{\mathrm{B}} T}{C_{\mathrm{l}}} D(E) \tag{26}$$

$$\frac{1}{\tau_{\mathrm{E,op}}} = \frac{\pi D_{\mathrm{O}}^2}{2\rho\omega_{\mathrm{o}}} \left[N_{\mathrm{o}} D(E + \hbar\omega_{\mathrm{o}}) + (N_{\mathrm{o}} + 1) D(E - \hbar\omega_{\mathrm{o}})\right] \tag{27}$$

where C_{l} is material's elastic constant, D_{A} is the acoustic deformation potential, D_{O} is the optical deformation potential (with unit [energy]/[length], different from what we used to plot Fig. 1d

because there we define deformation potentials along same high symmetry lines for easy comparison between acoustic and optical phonons), ρ is the mass density, ω_{o} is the optical phonon frequency at zone center, and N_{o} is the Bose-Einstein distribution function corresponding to this frequency. Combining these together we can calculate the electrical transport properties given by Supplementary Equations 24-25 and further evaluate the power factor (σS^2).

For acoustic phonon limited transport in the non-degenerate regime, one can obtain by plugging Supplementary Equation 26 into Supplementary Equations 24-25:

$$\sigma_{\mathrm{ac}} = \frac{2e^2}{3m^*}\frac{C_{\mathrm{l}}}{\pi D_{\mathrm{A}}^2}\mathrm{e}^{-\frac{E_{\mathrm{c}}-\mu}{k_{\mathrm{B}}T}} \tag{28}$$

$$S = \frac{k_{\mathrm{B}}}{e}\left(2 + \frac{E_{\mathrm{c}}-\mu}{k_{\mathrm{B}}T}\right) \tag{29}$$

which gives the power factor

$$\mathrm{PF}_{\mathrm{ac}} = \frac{2k_{\mathrm{B}}^2}{3m^*}\frac{C_{\mathrm{l}}}{\pi D_{\mathrm{A}}^2}\left[\mathrm{e}^{-\frac{E_{\mathrm{c}}-\mu}{k_{\mathrm{B}}T}}\left(2 + \frac{E_{\mathrm{c}}-\mu}{k_{\mathrm{B}}T}\right)^2\right] \tag{30}$$

where E_{c} is the electron energy at the conduction band edge. The optimal power factor is obtained by varying the Fermi level relative to E_{c} to maximize the term inside the bracket. This bracket only depends on the temperature, and therefore it is clearly seen that the dependence of the optimal power factor on the effective mass is only through the first term, namely, $\mathrm{PF}_{\mathrm{ac,opt}} \propto 1/m^*$. For optical phonon limited transport, we need to use Supplementary Equation 27. We propose the following approximation to simplify the expression in Supplementary Equation 27:

$$\frac{1}{\tau_{\mathrm{E,op}}} \approx \frac{\pi D_{\mathrm{O}}^2}{\rho\omega_{\mathrm{o}}}(N_{\mathrm{o}} + \lambda)D(E - \hbar\omega_{\mathrm{o}}) \tag{31}$$

where λ is between 0 and 1. In general a number of 0.5 will give a close estimation of the electron relaxation time due to optical phonon scattering. With Supplementary Equation 31, we can then calculate the transport properties, and the result yields (again for non-degenerate regime)

$$\sigma_{\mathrm{op}} = \frac{2e^2}{3m^*}\frac{2\rho\omega_{\mathrm{o}}k_{\mathrm{B}}T}{\pi D_{\mathrm{O}}^2}\frac{1}{N_{\mathrm{o}}+\lambda}\mathrm{e}^{-\frac{E_{\mathrm{c}}-\mu}{k_{\mathrm{B}}T}} \tag{32}$$

The Seebeck coefficient is the same as given by Supplementary Equation 29. The corresponding power factor is then described by

$$\mathrm{PF_{op}} = \frac{2k_\mathrm{B}^2}{3m^*}\frac{2\rho\omega_\mathrm{o}k_\mathrm{B}T}{\pi D_\mathrm{O}^2}\frac{1}{N_\mathrm{o}+\lambda}\left[\mathrm{e}^{-\frac{E_\mathrm{C}-\mu}{k_\mathrm{B}T}}\left(2+\frac{E_\mathrm{C}-\mu}{k_\mathrm{B}T}\right)^2\right] \tag{33}$$

It can be seen that the optimal power factor for optical phonon limited transport exhibits the same dependence on the effective mass as we have discussed for Supplementary Equation 30 under our approximation: $\mathrm{PF_{op,opt}} \propto 1/m^*$, thereby justifying the trend we see in Fig. 3c. In Supplementary Fig. 16 we also plot the power factors calculated directly using Supplementary Equation 27 without the assumption involved in deriving Supplementary Equations 31-32. We clearly see that the power factor with dominant optical phonon scattering indeed varies inversely proportional to the effective mass, as we have proved. We note that despite similar trend with respect to the effective mass, the power factor given by Supplementary Equation 33 is governed by entirely different scattering mechanism – the optical deformation potential can behave quite differently than the acoustic deformation potential regarding material structures, and may provide further opportunities for manipulating the electron scattering and therefore optimizing the electrical transport properties.

Supplementary References

1. Lambin, P. & Vigneron, J. Computation of crystal Green's functions in the complex-energy plane with the use of the analytical tetrahedron method. *Phys. Rev. B* **29,** 3430–3437 (1984).
2. Hess, K. *Advanced theory of semiconductor devices*. (Prentice-Hall, 1988).
3. M. Lundstrom. *Fundamentals of Carrier Transport*. (Cambridge University Press, 2009).
4. Gang Chen. *Nanoscale Energy Transport and Conversion: A Parallel Treatment of Electrons, Molecules, Phonons and Photons*. (Oxford University Press, 2005).
5. Giustino, F., Cohen, M. & Louie, S. Electron-phonon interaction using Wannier functions. *Phys. Rev. B* **76,** 165108 (2007).
6. Marzari, N., Mostofi, A. A., Yates, J. R., Souza, I. & Vanderbilt, D. Maximally localized Wannier functions: Theory and applications. *Rev. Mod. Phys.* **84,** 1419–1475 (2012).
7. Souza, I., Marzari, N. & Vanderbilt, D. Maximally localized Wannier functions for entangled energy bands. *Phys. Rev. B* **65,** 035109 (2001).
8. Qiu, B. *et al.* First-principles simulation of electron mean-free-path spectra and thermoelectric properties in silicon. *EPL Europhys. Lett.* **109,** 57006 (2015).
9. Li, W. Electrical transport limited by electron-phonon coupling from Boltzmann transport equation: An ab initio study of Si, Al, and MoS_2. *Phys. Rev. B* **92,** 075405 (2015).
10. Sjakste, J., Vast, N., Calandra, M. & Mauri, F. Wannier interpolation of the electron-phonon matrix elements in polar semiconductors: Polar-optical coupling in GaAs. *Phys. Rev. B* **92,** 054307 (2015).
11. Zhou, J.-J. & Bernardi, M. Ab initio electron mobility and polar phonon scattering in GaAs. *Phys. Rev. B* **94,** 201201 (2016).

12. Liu, T.-H., Zhou, J., Liao, B., Singh, D. J. & Chen, G. First-principles mode-by-mode analysis for electron-phonon scattering channels and mean free path spectra in GaAs. *Phys. Rev. B* **95,** 075206 (2017).

13. Wright, A. D. *et al.* Electron–phonon coupling in hybrid lead halide perovskites. *Nat. Commun.* **7,** 11755 (2016).

14. Park, C.-H. *et al.* Electron–Phonon Interactions and the Intrinsic Electrical Resistivity of Graphene. *Nano Lett.* **14,** 1113–1119 (2014).

15. Liao, B., Zhou, J., Qiu, B., Dresselhaus, M. S. & Chen, G. Ab initio study of electron-phonon interaction in phosphorene. *Phys. Rev. B* **91,** 235419 (2015).

16. Esfarjani, K. & Stokes, H. T. Method to extract anharmonic force constants from first principles calculations. *Phys. Rev. B* **77,** 144112 (2008).

17. Esfarjani, K., Chen, G. & Stokes, H. Heat transport in silicon from first-principles calculations. *Phys. Rev. B* **84,** 085204 (2011).

18. Carrete, J., Li, W., Mingo, N., Wang, S. & Curtarolo, S. Finding Unprecedentedly Low-Thermal-Conductivity Half-Heusler Semiconductors via High-Throughput Materials Modeling. *Phys. Rev. X* **4,** 011019 (2014).

19. Brooks, H. Theory of the Electrical Properties of Germanium and Silicon. in *Advances in Electronics and Electron Physics* (ed. Marton, L.) **7,** 85–182 (Academic Press, 1955).

20. Debye, P. & Hückel, E. Zur Theorie der Elektrolyte. I. Gefrierpunktserniedrigung und verwandte Erscheinungen. *Phys. Z.* **24,** 185–206 (1923).

21. Xie, H. *et al.* The intrinsic disorder related alloy scattering in ZrNiSn half-Heusler thermoelectric materials. *Sci. Rep.* **4,** 6888 (2014).

22. Fu, C., Zhu, T., Liu, Y., Xie, H. & Zhao, X. Band engineering of high performance p-type FeNbSb based half-Heusler thermoelectric materials for figure of merit zT>1. *Energy Env. Sci* **8,** 216–220 (2015).

23. Zhu, T. J. *et al.* Effects of yttrium doping on the thermoelectric properties of $Hf_{0.6}Zr_{0.4}NiSn_{0.98}Sb_{0.02}$ half-Heusler alloys. *J. Appl. Phys.* **108,** 044903 (2010).

24. Uher, C., Yang, J., Hu, S., Morelli, D. T. & Meisner, G. P. Transport properties of pure and doped MNiSn (M=Zr, Hf). *Phys. Rev. B* **59,** 8615–8621 (1999).

25. He, R. *et al.* Achieving high power factor and output power density in p-type half-Heuslers $Nb_{1-x}Ti_xFeSb$. *Proc. Natl. Acad. Sci.* **113,** 13576–13581 (2016).

26. Zhu, T. *et al.* Compromise and Synergy in High-Efficiency Thermoelectric Materials. *Adv. Mater.* **29,** 1605884 (2017).

27. Giustino, F. Electron-phonon interactions from first principles. *Rev. Mod. Phys.* **89,** 015003 (2017).

28. Baroni, S., de Gironcoli, S., Dal Corso, A. & Giannozzi, P. Phonons and related crystal properties from density-functional perturbation theory. *Rev. Mod. Phys.* **73,** 515–562 (2001).

29. Verdi, C. & Giustino, F. Fröhlich Electron-Phonon Vertex from First Principles. *Phys. Rev. Lett.* **115,** 176401 (2015).

30. Vogl, P. Microscopic theory of electron-phonon interaction in insulators or semiconductors. *Phys. Rev. B* **13,** 694–704 (1976).

31. Harrison, W. A. *Electronic structure and the properties of solids: the physics of the chemical bond*. (Dover Publications, 1989).

32. Ashcroft & Mermin. *Solid state physics*. (Cengage Learning, 1976).

33. Bassani, G. F. & Parravicini, G. P. *Electronic states and optical transitions in solids*. (Pergamon Press, 1975).
34. Inui, T., Tanabe, Y. & Onodera, Y. *Group Theory and Its Applications in Physics*. (Springer Science & Business Media, 2012).
35. Bir, G. L. & Pikus, G. E. *Symmetry and strain-induced effects in semiconductors*. (Wiley, 1974).
36. Koster, G. F. *Properties of the thirty-two point groups*. (M.I.T. Press, 1963).
37. Cotton, F. A. *Chemical Applications of Group Theory*. (Wiley, 1994).
38. Miessler, G. L. & Tarr, D. A. *Inorganic Chemistry*. (Pearson Prentice Hall, 2004).
39. Mulliken, R. S. Electronic Population Analysis on LCAO–MO Molecular Wave Functions. I. *J. Chem. Phys.* **23,** 1833–1840 (1955).
40. Mulliken, R. S. Electronic Population Analysis on LCAO–MO Molecular Wave Functions. II. Overlap Populations, Bond Orders, and Covalent Bond Energies. *J. Chem. Phys.* **23,** 1841–1846 (1955).
41. McQuarrie, D. A. *Quantum Chemistry*. (University Science Books, 2008).
42. Harrison, W. A. *Elementary Electronic Structure*. (World Scientific, 1999).
43. A comparative study of Hamilton and overlap population methods for the analysis of chemical bonding. *J. Chem. Phys.* **113,** 1698–1704 (2000).
44. Hoffmann, R. *Solids and Surfaces: A Chemist's View of Bonding in Extended Structures*. (VCH Publishers, 1988).
45. Hughbanks, T. & Hoffmann, R. Chains of trans-edge-sharing molybdenum octahedra: metal-metal bonding in extended systems. *J. Am. Chem. Soc.* **105,** 3528–3537 (1983).

46. Dronskowski, R. & Bloechl, P. E. Crystal orbital Hamilton populations (COHP): energy-resolved visualization of chemical bonding in solids based on density-functional calculations. *J. Phys. Chem.* **97,** 8617–8624 (1993).

47. Löwdin, P. On the Non‐Orthogonality Problem Connected with the Use of Atomic Wave Functions in the Theory of Molecules and Crystals. *J. Chem. Phys.* **18,** 365–375 (1950).

48. Maintz, S., Deringer, V. L., Tchougréeff, A. L. & Dronskowski, R. Analytic projection from plane-wave and PAW wavefunctions and application to chemical-bonding analysis in solids. *J. Comput. Chem.* **34,** 2557–2567 (2013).

49. Deringer, V. L., Tchougréeff, A. L. & Dronskowski, R. Crystal Orbital Hamilton Population (COHP) Analysis As Projected from Plane-Wave Basis Sets. *J. Phys. Chem. A* **115,** 5461–5466 (2011).

50. Zhou, J., Liao, B. & Chen, G. First-principles calculations of thermal, electrical, and thermoelectric transport properties of semiconductors. *Semicond. Sci. Technol.* **31,** 043001 (2016).

51. Slack, G. New Materials and Performance Limits for Thermoelectric Cooling. in *CRC Handbook of Thermoelectrics* (CRC Press, 1995). doi:10.1201/9781420049718.ch34

52. Caillat, T., Borshchevsky, A. & Fleurial, J. ‐ P. Properties of single crystalline semiconducting $CoSb_3$. *J. Appl. Phys.* **80,** 4442–4449 (1996).

53. LaLonde, A. D., Pei, Y. & Snyder, G. J. Reevaluation of $PbTe_{1-x}I_x$ as high performance n-type thermoelectric material. *Energy Environ. Sci.* **4,** 2090–2096 (2011).

54. Pei, Y., LaLonde, A., Iwanaga, S. & Snyder, G. J. High thermoelectric figure of merit in heavy hole dominated PbTe. *Energy Environ. Sci.* **4,** 2085–2089 (2011).

55. Zhang, Q. *et al.* Enhancement of Thermoelectric Performance of n-Type PbSe by Cr Doping with Optimized Carrier Concentration. *Adv. Energy Mater.* **5,** n/a-n/a (2015).

56. Muta, H., Kanemitsu, T., Kurosaki, K. & Yamanaka, S. High-temperature thermoelectric properties of Nb-doped MNiSn (M=Ti, Zr) half-Heusler compound. *J. Alloys Compd.* **469,** 50–55 (2009).

57. Mao, J. *et al.* Thermoelectric Properties of n-type ZrNiPb-Based Half-Heuslers. *Chem. Mater.* **29,** 867–872 (2017).

58. High-Thermoelectric Figure of Merit Realized in p-Type Half-Heusler Compounds: $ZrCoSn_xSb_{1-x}$. *Jpn. J. Appl. Phys.* **46,** L673 (2007).

59. Rausch, E., Balke, B., Ouardi, S. & Felser, C. Enhanced thermoelectric performance in the p-type half-Heusler (Ti/Zr/Hf)$CoSb_{0.8}Sn_{0.2}$ system via phase separation. *Phys. Chem. Chem. Phys.* **16,** 25258–25262 (2014).

60. He, R. *et al.* Improved thermoelectric performance of n-type half-Heusler $MCo_{1-x}Ni_xSb$ (M = Hf, Zr). *Mater. Today Phys.* **1,** 24-30 (2017).

61. Ponnambalam, V., Zhang, B., Tritt, T. M. & Poon, S. J. Thermoelectric Properties of Half-Heusler Bismuthides $ZrCo_{1-x}Ni_xBi$ (x = 0.0 to 0.1). *J. Electron. Mater.* **36,** 732–735 (2007).

62. He, R. *et al.* Enhanced thermoelectric properties of n-type NbCoSn half-Heusler by improving phase purity. *APL Mater.* **4,** 104804 (2016).

63. Oestreich, J., Probst, U., Richardt, F. & Bucher, E. Thermoelectrical properties of the compounds $ScM_{VIII}Sb$ and $YM_{VIII}Sb$ (M_{VIII} = Ni, Pd, Pt). *J. Phys. Condens. Matter* **15,** 635 (2003).

64. Li, S., Zhao, H., Li, D., Jin, S. & Gu, L. Synthesis and thermoelectric properties of half-Heusler alloy YNiBi. *J. Appl. Phys.* **117,** 205101 (2015).

65. Kimura, Y., Kuji, T., Zama, A., Lee, T. & Mishima, Y. Thermoelectric Properties of Half-Heusler Compounds n-type MNiSn and p-type MPtSn (M = Hf, Zr). *MRS Online Proc. Libr. Arch.* **980,** (2006).

66. Blöchl, P. E. Projector augmented-wave method. *Phys. Rev. B* **50,** 17953–17979 (1994).

67. Yu, C. *et al.* High-performance half-Heusler thermoelectric materials $Hf_{1-x}Zr_xNiSn_{1-y}Sb_y$ prepared by levitation melting and spark plasma sintering. *Acta Mater.* **57,** 2757–2764 (2009).
